# Addressing standardization and semantics in an electronic lab notebook for multidisciplinary use: LabIMotion

**DOI:** 10.1186/s13321-025-01021-4

**Published:** 2025-05-14

**Authors:** Chia-Lin Lin, Pei-Chi Huang, Christof Wöll, Patrick Théato, Christian Kübel, Lena Pilz, Nicole Jung, Stefan Bräse

**Affiliations:** 1 Institute of Biological and Chemical Systems, Functional Molecular Systems (IBCS), Karlsruhe Institute of Technology, Kaiserstraße 12, 76131 Karlsruhe, Germany; 2 Institute of Functional Interfaces (IFG), Karlsruhe Institute of Technolgy, Kaiserstraße 12, 76131 Karlsruhe, Germany; 3 Institute for Chemical Technology and Polymer Chemistry (ITCP), Karlsruhe Institute of Technology, Kaiserstraße 12, 76131 Karlsruhe, Germany; 4 Soft Matter Synthesis Laboratory, Institute for Biological Interfaces III (IBG3), Kaiserstraße 12, 76131 Karlsruhe, Germany; 5 Institute of Nanotechnology (INT), Karlsruhe Institute of Technology, Kaiserstraße 12, 76131 Karlsruhe, Germany; 6 Karlsruhe Nano Micro Facility (KNMFi), Karlsruhe Institute of Technology, Kaiserstraße 12, 76131 Karlsruhe, Germany; 7 Institute of Organic Chemistry, Karlsruhe Institute of Technology, Kaiserstraße 12, 76131 Karlsruhe, Germany; 8 In-situ Electron Microscopy, Technical University Darmstadt, Peter-Grünberg-Straße 2, 64287 Darmstadt, Germany

**Keywords:** Electronic lab notebook, FAIR data, Data transfer, Digitalization, Chemistry

## Abstract

This work presents the LabIMotion extension for the Chemotion Electronic Lab Notebook (ELN), expanding its capabilities from organic chemistry to support interdisciplinary research and enabling the description of workflows. LabIMotion enhances documentation by introducing customizable components structured across three levels—*Elements*, *Segments*, and *Datasets*—enabling flexible, hierarchical organization and reuse of data. Through the integration of links to ontologies, the extension ensures precise, machine-readable data, promoting interoperability and adherence to FAIR principles. The extension features an intuitive, user-friendly interface that allows researchers to easily create new ELN content by leveraging a set of customizable, generic methods. Scientists can set up new data fields, can link data fields, or establish workflows, and the extension translates those needs directly into usable functionality at their command. Through this high degree of flexibility, a wide range of specific research needs can be met. The LabIMotion Hub plays a crucial role in distributing and updating components, fostering standardization, and enabling collaborative development within scientific communities. These advancements significantly improve the ELN's adaptability, usability, and relevance across various research disciplines.

**Scientific contribution**

This work demonstrates how research data management systems can be designed to support discipline-specific requirements in chemistry research while offering a high flexibility and interoperability to deal with interdisciplinary work. The developed software, LabIMotion, offers a versatile approach for integrating novel research aspects into a research data environment, fostering bottom-up processes for defining schemas and standardizing scientific workflows. In particular, the software’s support for community-driven extensions, combined with a clear definition of content and its assignment to ontology terms, provides unique advantages for creating adaptable tools suited to the complexities of the scientific environment.

**Supplementary Information:**

The online version contains supplementary material available at 10.1186/s13321-025-01021-4.

## Introduction

Electronic Lab Notebooks (ELNs) play a pivotal role in digitalizing laboratory procedures, supporting the improvement of experiment reproducibility and fostering transparent, traceable scientific practices with ease [1]. ELNs can support the generation of FAIR (Findable, Accessible, Interoperable, Re-usable) data [2] which offers key benefits in comparison to the manual tracking and documentation of research processes [3,4]. Also, more and more communities become aware of the limited number of well-structured data being available for the development of AI methods—which hinders efficient data analysis in order to accelerate the scientific development in experimental sciences [5]. Therefore, ELNs have evolved into essential tools for contemporary research facilities, streamlining data management, fostering collaboration, and documenting scientific endeavors. Notably, ELNs are endorsed by numerous funding bodies and communities for their sustainability and reproducibility benefits [6,7], leading to a growing uptake within academic circles [1,8,9]. There are different types of ELNs in use. Generic or multidisciplinary ELNs typically support a discipline-agnostic approach, providing researchers with significant freedom in data storage methods and process documentation [10,11]. While generic ELNs can be used across various scientific domains, they are often limited in providing well-annotated, structured and standardized data. Depending on the requirements of a specific discipline, limitations may arise, impacting the efficiency of scientific processes, the searchability and the reusability of results due to less-structured data. Furthermore, there is a multitude of Electronic Lab Notebooks (ELNs) with specialized tools and a data structure well adapted to each respective discipline [12,13]. The standards and processes prescribed by these discipline-specific ELNs are defined to effectively support and accelerate scientific work, and discipline specific ELNs prepare data storage in a suitable manner to ensure FAIR data. This translates to good direct reusability of the data and potentially prepares the data for long-term reusability and comparability with other data sources.

Unfortunately, those ELNs that offer very good structuring and adaptation to requirements from a discipline-specific perspective are usually not universally applicable and therefore not suitable for all applications. As a consequence, this means that interdisciplinary work cannot usually be well supported by discipline-specific ELNs which is hindering the description of e.g. workflows that are an important part of the work in multidisciplinary research teams. These ELNs also find less acceptance in the wider community, as larger infrastructures such as university computer centers are usually unable to provide discipline-specific support in research data management, and don't want to support and administer multiple different software systems.

making it difficult to host various specialized ELNs via central facilities. An overview of frequently used ELNs at present can, for example, be obtained through the service ELNFinder. [14]

## Implementation

In this work, we present a way to extend a previously organic chemistry-focused ELN, Chemotion ELN, so that it can also be used for interdisciplinary work and in disciplines beyond the originally supported field. Chemotion [12] is an open-source ELN developed within the National Research Data Infrastructure of Chemistry (NFDI4Chem) [15,16], providing features to facilitate data management, experiment documentation, and the analysis of measurements and analytical data [17]. It further includes a variety of options to connect devices and to manage data transfer and data storage [18]. Chemotion offers tools for recording experimental data, for drawing and processing chemical structures [19,20], for transforming proprietary files into open file formats, for the management of a chemical inventory, and for the organization of research workflows. It can be used to seamlessly transfer data into the research data repository Chemotion [21] to publish research data. In the version as described, Chemotion offers advanced documentation and analysis options for chemists, in particular experimental chemists dealing with small to medium sized molecules. However, in discussions with collaborators that intended to use Chemotion interdisciplinary projects that go beyond chemistry, several missing functions were identified. The requirements were collected in the form of three use-cases:

1. Extension for Polymer Chemistry: Polymer chemistry as part of the domain of chemistry requires with respect to documentation (e.g. reaction) and analysis (e.g. nuclear magnetic resonance, NMR) a lot of functions that correspond to those of molecular chemists (e.g. organic and inorganic chemistry). Nevertheless, the polymer chemists need to record several characteristics in particular for polymer samples that are usually not described for standard chemistry samples. The analytical description of the samples requires additional analytical techniques such as thermogravimetric analysis (TGA), digital scanning calorimetry (DSC) and size exclusion chromatography (SEC).
2. Reactions, samples and analyses for Metal organic frameworks: Metal organic frameworks (MOFs) thematically bridge aspects of chemistry with materials sciences. Making an ELN suitable for science with MOFs, a suitable description of the preparation of MOFs and their description in the form of samples including analytical data is required. The analytical data section needs in particular an extension allowing the representation of single crystal X-ray diffraction (SCXD) and powder X-ray diffraction (PXRD).
3. Workflows for TEM and SEM sample preparation: the description of TEM and SEM measurements which are used for the investigation of samples in many disciplines, require a detailed documentation on the preparation of the samples in the form of a workflow containing details about different processing steps that are conducted with the sample.

With LabIMotion, an extension to Chemotion, we pursue four main goals referring to a multidisciplinary use of the ELN in the future. The goals are a combination of different needs which were abstracted from the use cases and were combined with general requirements for research data infrastructures. [1,3,22]

Goal 1: Documentation of research work on a broad scale: We aim to bridge the gap that currently exists between the use of either specifically tailored and discipline-agnostic ELNs. In particular, we intend to provide a documentation tool that supports all research aspects and disciplines related to chemistry. While a wide range of applications will be supported, those with a connection to chemistry will particularly benefit from the existing functions designed for chemical research.

Goal 2: Standardization, comparability, reproducibility: The proposed implementation aims to enable a high level of standardization across all areas of documentation, allowing scientists working in the same research fields to make equivalent entries in their documentation tools. Wherever possible, the comparability of these entries should be supported, thereby also achieving a high level of reproducibility in the research.

Goal 3: Machine-readable and interpretable data: The ELN extension aims to enable highly detailed and structured data entries, supported by defined vocabularies and ontologies in various sections as a basis for a later reuse independent of human interpretation. The extension should work beyond the domain of chemistry [23] and it should allow the semantic enrichment of data without tagging as used in former approaches [24]. The direct implementation of semantic description should avoid the use of external software to extract and map metadata [25].

Goal 4: FAIR data: The implementation is designed to technically support the creation of FAIR data, as much as possible within the established processes and definitions of scientific work.

Achieving these goals requires the development of components to technically extend the Chemotion ELN. Additionally, both short-term and long-term engagement of various scientific communities are necessary. These communities will define the content of the documentation areas, establish relevant links to existing modules, and enable continuous improvement of the workspaces through extensions and adjustments.

### Basic concepts enable the definition of different levels and their combination

The ELN extension offers various methods for structuring information, distinguishing between three levels: processes, workflows and entities (hereafter referred to as *Elements*), detailed descriptions of entities (hereafter referred to as *Segments*), and measurement data descriptions (hereafter referred to as *Datasets*). The given structure is a result of the need to store descriptions of processes, workflows and entities (- > Elements) and assigned data (- > Datasets) in many scientific domains. Many available ELNs support these structural components as a minimum requirement [10,11,26,27], the LabIMotion extension additionally offers Segments as an option to modularly extend Elements (for further information, see SI Sect. 1).

**Fig. 1:**
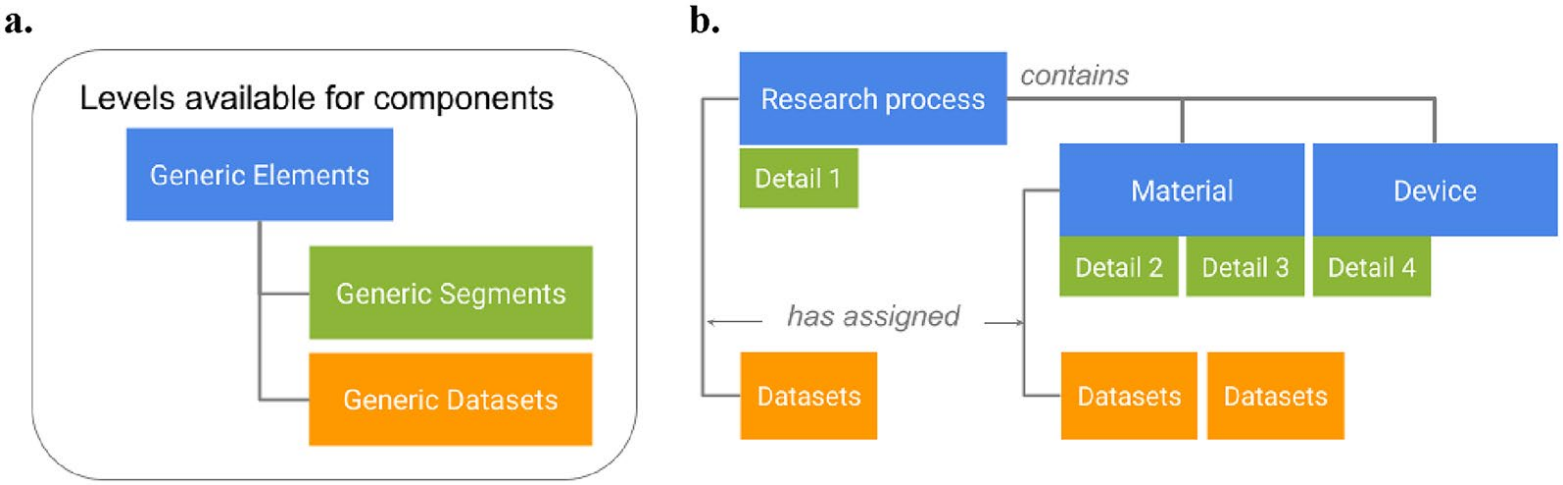
Main structural components available for the components that can be designed with the LabIMotion extension: **a** three different components types can be designed independent of each other and combined in a flexible manner. **b** An example implementation for the use of *Elements* (e.g. research process, material, device), *Segments* (e.g. detail 1 to detail 4), and *Datasets*. The levels correspond to the currently available levels in the main Chemotion implementation. E.g. in Chemotion exist “Samples” and “Reactions” (= *Elements*), additional information to Samples (= Segments) and Datasets (= Datasets), being therefore interoperably usable with the new LabIMotion extensions

The predefinition of the three levels and their relation to each other facilitates the generation of structured data and the standardization of work processes which is required to reach goal 2 in this work. For a flexible organization of ELN content serving the needs of different use cases (to reach goal 1) and effective reuse of descriptions, the following principles apply:

A. Independent definition of levels: The levels can each be defined independently, allowing for iterative expansion in various design processes. The independent development of level components is a key requirement for the generalized use of individual components and the ability to combine them in a flexible manner.
B. Use of levels in a hierarchical structure: The levels are organized hierarchically. Processes and single entities are described as *Elements* in the form of basic information. More detailed descriptions, which may apply to different sub-types of processes or entities, are categorized as *Segments* and associated with the respective *Elements*. *Datasets* are described as measurement data records related to *Elements* (Fig. 1)
C. Referencing of components: Further structuring of ELN content can be achieved through referencing. For example, *Elements* such as processes or workflows can be further described by the referencing and linking of other *Elements* (for additional examples, see SI).
D. Flexible combination of individual components: The design described above allows for diverse combinations of components. For instance, (a) an *Element* can be associated with any other *Element* or *Segment*, (b) one or more *Segments* can be used to describe one or more *Elements*. (c) *Datasets*, as part of measurement data, can be combined with all types of *Elements* and can be assigned to stages within *Segments* and *Elements*. If workflows are designed, the combination of different *Elements* within a workflow can result in a whole collection of referenced and structured *Elements*.

### Development and implementation of methods

An important aspect of expanding the ELN is providing methods that allow users without programming knowledge to integrate their documentation needs into the ELN. This approach requires more programming effort from the developers compared to coding a specific use case and necessitates excellent coordination with potential ELN users. Specific requirements must be translated into generally applicable methods that can be used in a wide range of applications, and then these methods must be provided. However, once these requirements have been implemented, the resulting generic methods can be broadly used by all users and enable the provision of a wide range of functions, field types, and UI customizations. The methods are generally developed for all levels of ELN expansion but must be adapted to specific requirements and dependencies.

### GUI-supported use of the methods

**Fig. 2:**
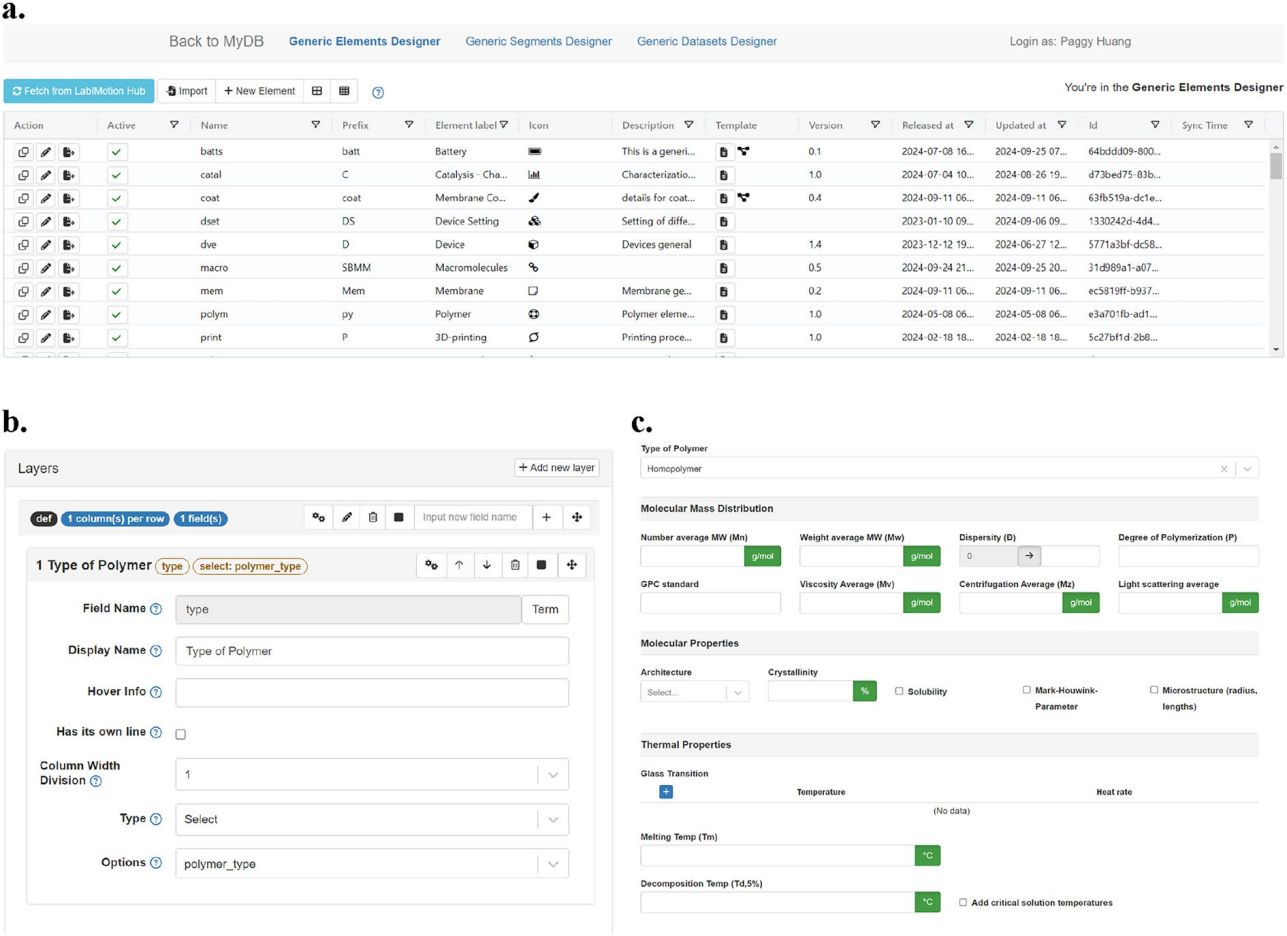
**a** Screenshot of the management areas of an *Element* designer, listing the started and finished *Elements* and offering diverse functions for their use, further editing and versioning; **b** screenshot covering a selection of options in the work area for the definition of *Segments* with the offered generic methods; **c** screenshot of the preview area of the *Segment* designer, showing how the methods are implemented in the final *Segment*. While a. and b. describe the way how to design new LabIMotion components, c. visualizes how the results are integrated into the Chemotion framework.

**Fig. 3:**
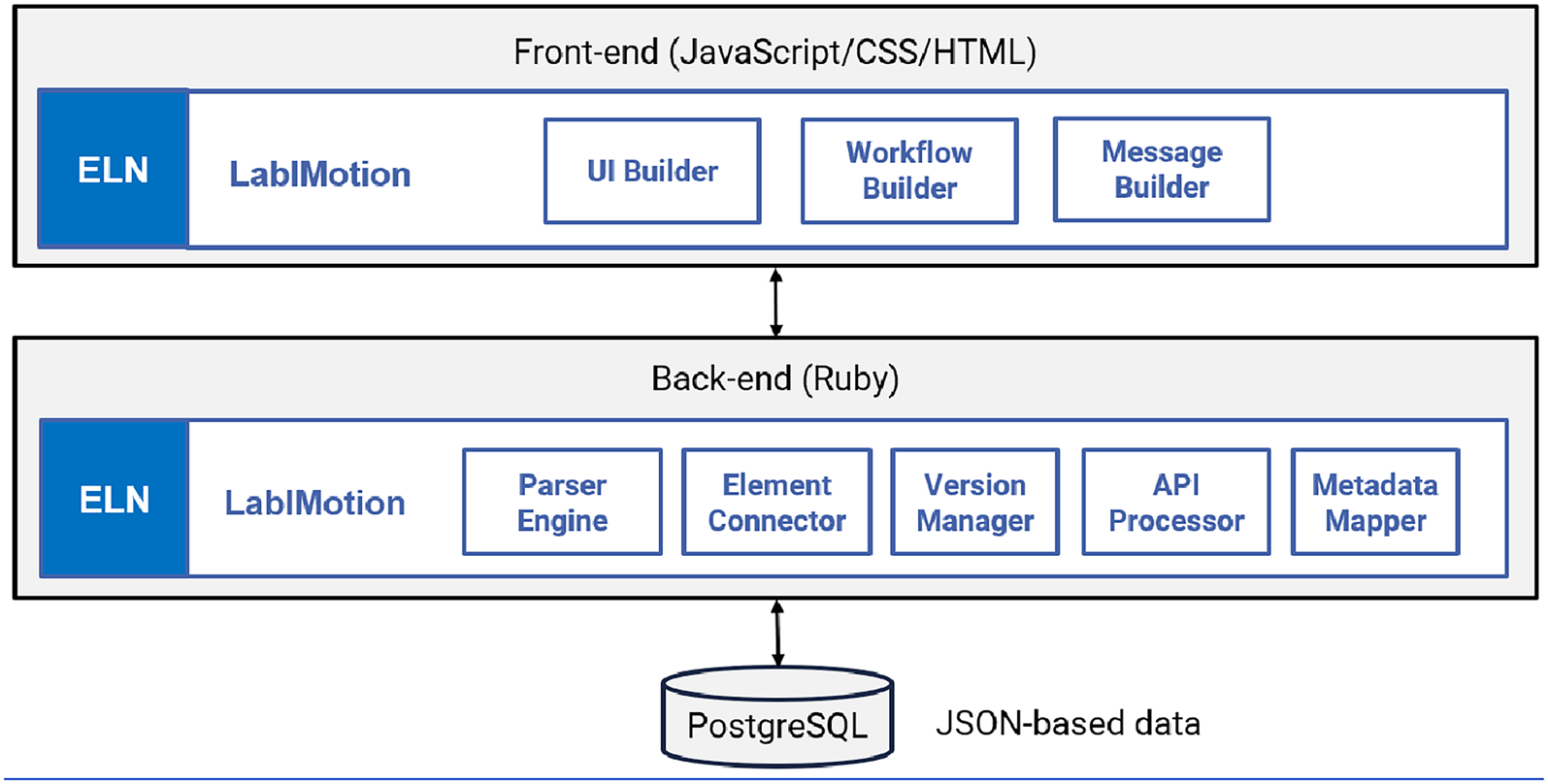
The diagram delineates the architectural structure of LabIMotion. The extension possesses its own distinct components which can be dissected into a front-end, a backend and a database layer. Front-end and back-end combine different modules to ensure the desired functionality. The front-end components integrate LabIMotion into the Chemotion framework without any visual difference referring to the Chemotion chemistry features provided by default

The methods provided by the LabIMotion extensions are made easily understandable and accessible to users through a Graphical User Interface (GUI) (Fig. 2). The GUI was developed using JavaScript, CSS, and HTML programming languages and technically uses three modules for its functionality: a UI builder to craft the user interface, a workflow builder for the definition and management of workflows, and a message builder for the creation and management of system messages (Fig. 3). The GUI distinguishes between the different levels for the hierarchical structure of the ELN, which include the three component types: *Elements*, *Segments*, and *Datasets*. For each of these three types, the GUI offers distinct design areas. Each area contains a management section, a workspace, and a preview designer (Fig. 2). The management area gives an overview of the components that have already been started or established. It allows insight into all components that are available to the user and provides special functions to organize them, including the editing of metadata, the activation and deactivation in the productive ELN environment, and the download for sharing (example for *Elements*: Fig. 2a). The work area provides the main functionality to structure the components, to assign the desired functionality and to design the UI (example for *Segments*: Fig. 2b). The changes can be saved as different versions, allowing a clear development strategy including the rebase to previous versions if required. In parallel to the development of the components in the work area, a preview area can be accessed, showing the current layout as a result of the work area. The preview area supports checking the basic functionality to ensure the proper implementation of the desired functions and dependencies (example for *Segments*: Fig. 2c).

### Backend process and storage

In the whole Chemotion software, including the LabIMotion extension, the front-end interacts with the back-end, which subsequently interacts with the Data Storage. The back-end represents the server-side of the system and is developed in Ruby. For enabling the LabIMotion extension, the backend includes five main components: (1) The **Parsing Engine** is responsible for breaking down and interpreting raw data into structured information, handling the initial processing of the data and converting it into a format that can be further processed. (2) The **Element Connector** can be tasked with linking different system elements. It establishes and maintains relationships between various entities to ensure that proper associations are created. (3) The **Version Manager** tracks and manages different versions of data over time. LabIMotion keeps a history of changes made to the data and creates and saves new versions based on user actions. A key feature is the ability to revert to a specific version. (4) The **API Processor** is responsible for managing interactions between different parts of the system through the API (Application Programming Interface). It handles the request process, response handling, and data exchange, providing a centralized way to handle the logic associated with API requests and responses between clients and servers, as well as consistent error handling. (5) Finally, the **Metadata Mapper** ensures that metadata is mapped between different data structures and formats and handles the necessary conversions. The data storage layer uses PostgreSQL, an advanced open-source relational database that can accommodate both standard structured data and JSON-based data. Structured data is organized using normalization principles to reduce redundancy and ensure data integrity and to maintain structured data relationships. JSON-based data is used to enforce the storage of differently structured data and to handle complex data structures such as nested objects and arrays. With this dual strategy, LabIMotion can take advantage of the strengths of each type to handle a wide variety of data scenarios, and PostgreSQL adheres to the ACID (Atomicity, Consistency, Isolation, Durability) principle to ensure reliable transactions. This means that operations on both structured and JSON-based data maintain consistency and integrity.

## Results

The contents of individual components can be structured independently of the chosen level of the component by fields and layers (allowing for the repetition of content consisting of multiple fields). Additionally, for the *Elements* level, there is the option to define workflows. The use of generic methods being applied to different scenarios independent of the discipline allows us to reach goal 1 of our work, the support of documentation of research on a broad scale. The predefinition of layers and fields to be reused by others supports the need for standardization, and comparability as outlined in goal 2.

**Fig. 4.**
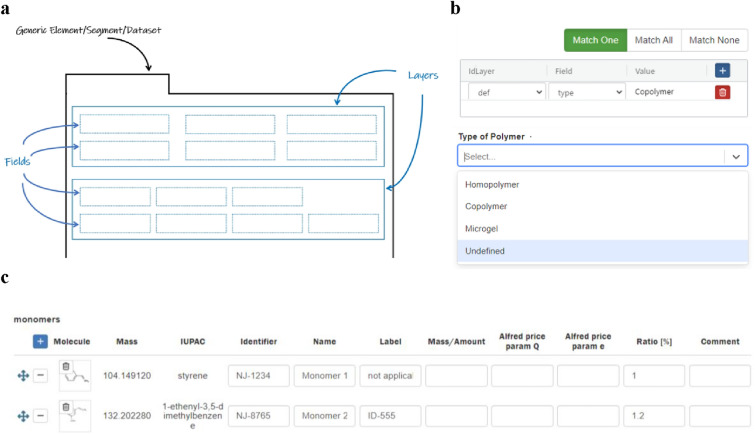
**a** Schematic view describing the combination of field and layers as structural and design parts of all *Elements*, *Segments* and *Datasets*; **b** exemplarily chosen application of dependencies that allow the definition of complex scenarios; **c** representation of a table in a LabIMotion component allowing the summary of manifold items with the same types of information in a condensed manner. Single fields and tables can include drag and drop options to link data across different ELN content areas. Due to the deep integration of the LabIMotion components into Chemotion, drag and drop options can be used for Chemotion and LabIMotion components without any difference

Methods and options for layers: Layers represent the highest organizational unit within a component, grouping together various input fields (Fig. 4a). They provide a framework for a collection of information that is visually unified, can have a title, and are collectively subject to dependencies. Layers can be timestamped, repeated as a unit, and assigned to datasets to clearly associate measurement data with different stages in a scientific process.

Methods and options for fields in layers: Fields offer a wide range of possibilities regarding their visual integration into the UI and the implemented functionality. In the current stage of LabIMotion development, a total of 17 field types are available (see SI, Sect. 4), each with its own UI design. Additionally, the creator of the components can define their positioning within the UI. Alongside functionally, simple field types like text fields, select boxes, and fields that combine values and units, more complex field types are also supported. These include tables, form fields, drag-and-drop fields, and selected combinations of these fields (Fig. 4b). To all fields, different dependencies can be assigned, very similar to the options for layers. This allows the implementation of a complex behavior of the UI depending on the required information type, detail level and complexity, making the ELNs content adaptable to the scientific setting by the ELN user. While the overall design of components can already be predefined through layers, a detailed assignment of the position and size of individual input fields is also possible. This is particularly important for fields that, due to either a complex design (e.g., tables) or the anticipated volume of information, should not fall below a certain size. Fields can be defined with a proportional width or can be applied across the full width of the interface.

**Fig. 5:**
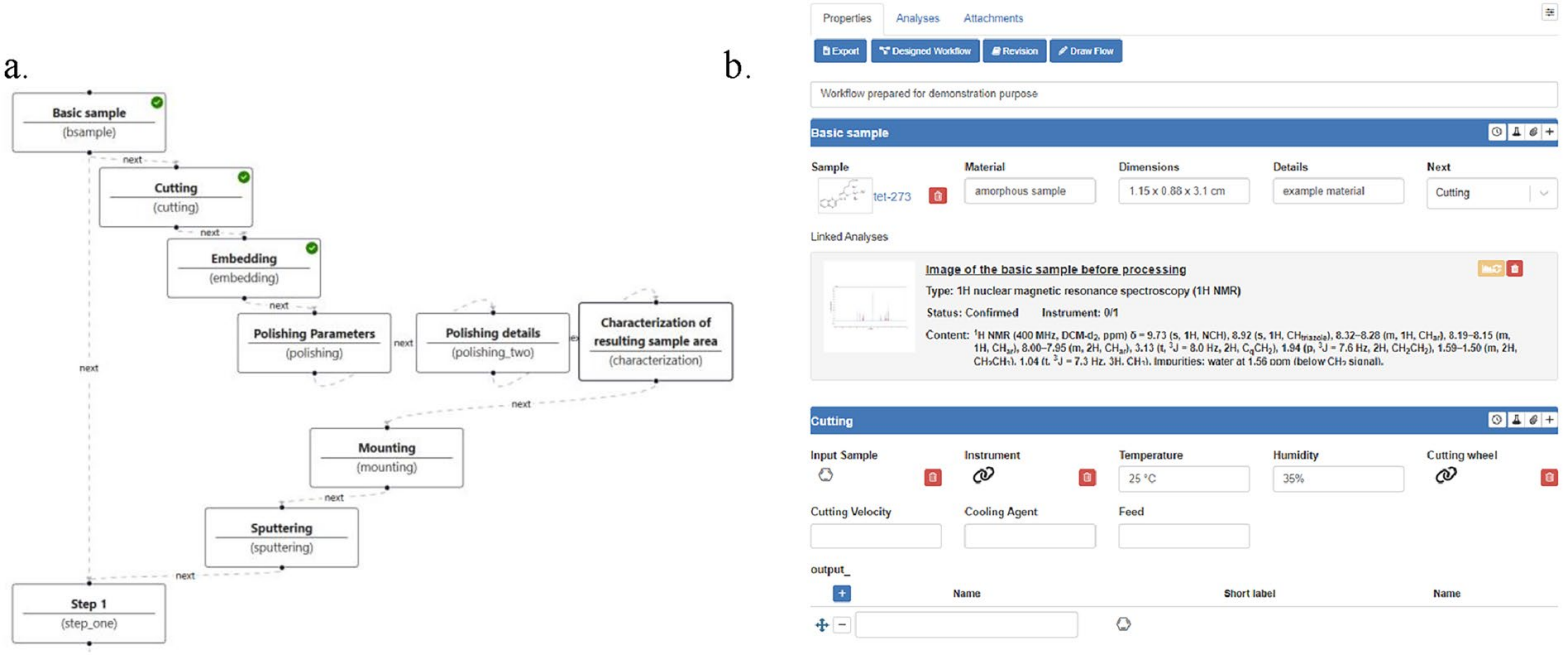
Example for the design of a workflow scheme in LabIMotion. **a** The overview panel of the workflow shows the overall available steps—each consisting of a layer—with the already used layers with added information given with a green mark; **b** Detailed view of the input fields and options available within the selected layers of the workflow. Symbols within the layers represent the drag and drop areas (e.g. for samples or instruments) that need the link of a related item from other ELN environments

Methods and options for workflows: The design of workflows is supported at varying levels of detail. Different options for strict or flexible guidance through the workflows can be combined. Content-wise, workflows are defined using the same methods available through layers and fields. Within workflows, the creator of a component can also define the sequence of various processes, thereby suggesting to the user a sequence of actions and their options. These sequences can, but do not have to, be defined through tree structures. The workflow options allow the repetition of layers and, if needed, the design of workflows that are not based on predefined scenarios, but allow the self-defined selection of a certain number of layers defined for the workflow. This concept enables the flexible design including the combination of predefined sub-units in the form of facultatively applicable layers (Fig. 5).

### Implementation of terminologies and ontologies

**Fig. 6:**
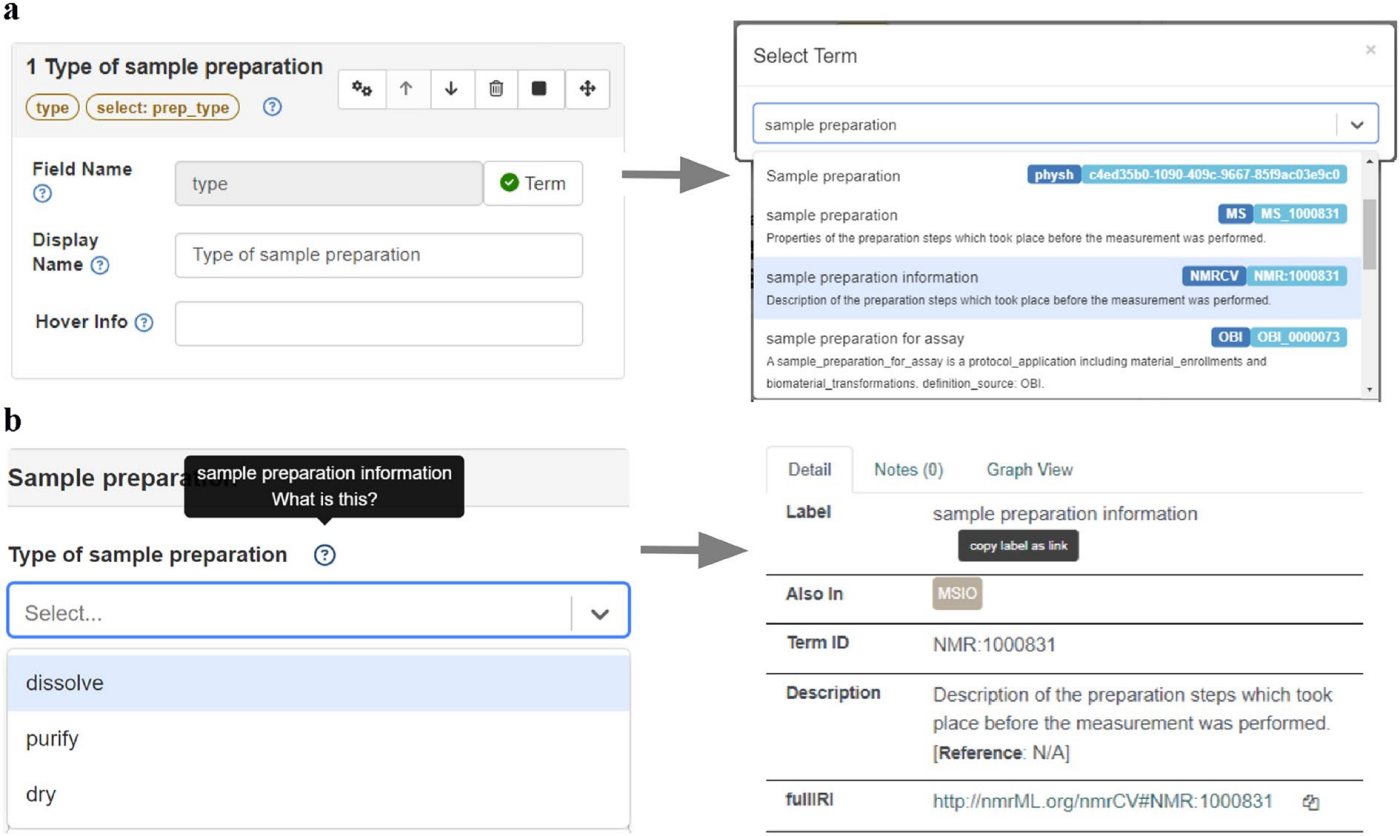
Example for an input field (here: “Type of sample preparation”) in a dataset component for which the assignment of an ontology term is shown. **a** Generation of a new field (**a**, left) and definition of its name in the LabIMotion GUI as well as query of TIB’s ontology service (directly available from the ELN’s interface) to identify a suitable term for the new input field (**a**, right). **b** After the assignment of a term, the input field combines information on the meaning of the field type with the select options for the “type of sample preparation” (**b**, left). If information on the meaning is required, the direct link to the ontology service gives more details and a clear definition (**b**, right)

To ensure the precise description of new ELN areas implemented through methods and to enhance the reusability of the data outside the ELN environment, individual input fields and component areas can be completed with terminologies from ontologies (Fig. 6). The further semantic description of templates up to a level of single input fields contributes to goal 3 and goal 4 of our work. Two principal approaches have been implemented: **On the Datasets Level:** The developed components must be defined using terms from an appropriate methods ontology. This allows for a clear description of the implemented procedures and provides a systematic delivery of the correct metadata forms based on the analytical measurement methods performed within the ELN environment. **For All Levels (Datasets, Segments, and Elements):** The assignment of an unlimited number of terms from any vocabularies and ontologies available through the TIB's terminology service [28] is supported. Since the application areas and thus the required terms can vary widely, the system does not impose restrictions on the choice of terms. When a term is present in multiple terminologies, the creator of an ELN component is responsible for selecting the appropriate description.

### Application of new ELN components

The creation of new components (design) is generally possible for all users, provided they have been granted the necessary permissions by the system administrator. The newly generated components can be applied in an ELN environment either by the creator of the components or by the admin, who can make the new components available to all users. Within an ELN instance, this is done through the management function of the respective levels by changing the component's status from deactivated to activated. Once activated, *Elements* and *Segments* are optionally made available to users on the ELN interface and can be used in combination with the core modules of the Chemotion ELN. *Datasets* become automatically available when measurement data is created and the corresponding ontology terms are selected. If definitions for data extraction are present [29], the metadata of the measurement data is extracted from the data files and assigned to the fields of the datasets, meaning that the forms are automatically populated using the measurement data as the source of information. Sharing the created components beyond one's own instance is facilitated through download and upload options available for all components. Additionally, components can be proposed for central distribution via the LabIMotion Hub (see the following chapter).

### Strategy for standardized components and sustainability

The approach to creating new components for an ELN, as outlined in this publication, focuses on the technical possibilities and their implementation within the ELN areas. These capabilities (contributing to goal 1) allow even an individual user to design and activate new components for personal use, thereby creating an optimal working and documentation environment tailored to their specific needs. While this can be a useful solution in certain cases to quickly adapt the ELN to individual requirements, the technology should, whenever possible, be used by one or more groups of scientists who collaboratively discuss, agree on, and implement the needs of their communities. Ideally, at least one person from the ELN development team should be involved in an advisory capacity during the design process. This ensures that the systematic structure of existing ELN components, along with their relationships and dependencies, is understood and can be taken into account when planning new components. For the correct assignment of ontologies, the consultation of an ontology expert is recommended, ensuring that basic semantic rules are considered. To facilitate the use and distribution of components created through the consensus of a larger group of scientists, the LabIMotion Hub (see also SI, Sect. 3) was established^30^. The Hub is an openly accessible service which enables the provision of newly generated components to other users and ensures that the latest version of both existing and updated components is available. New and updated versions of designed ELN components can be obtained through the Hub and directly updated and used within the user's own ELN environment. The Hub plays a crucial role in the central distribution of available components, regardless of the creators' group. It serves as a means to reach goal 2 of our work by promoting standardization in the description of information across various scientific groups and ELN instances, as well as to systematically track and evaluate ongoing expansions. In cases where the Hub is not considered to be suitable to exchange templates, they can also be exported from one ELN instance and imported to a second one.

## Conclusion

In this work, we present an approach for extending the Chemotion Electronic Lab Notebook, originally specialized in organic chemistry, to support interdisciplinary research across various scientific fields including complex workflow settings. The LabIMotion extension aims to create a versatile and user-friendly documentation tool that not only addresses the specific needs of chemical research but also benefits a broader range of scientific disciplines. Our objectives include facilitating comprehensive research documentation, promoting standardization, comparability, and reproducibility, and ensuring that data is machine-readable and adheres to FAIR principles. To achieve these goals, we have developed a flexible system of components that allows users to structure information across three levels: *Elements*, *Segments*, and *Datasets*. The design of these components is highly customizable, offering a hierarchical structure and the ability to reference other components, thus enhancing the flexibility and reusability of the ELN content. Through the integration of terms linked to ontologies and vocabularies, components are enriched with precise terminology, improving data interoperability and reuse beyond the ELN environment. The creation and implementation of new components can be carried out by any user with the necessary permissions, allowing for rapid adaptation to individual needs. However, we emphasize the importance of collaborative component development within scientific communities to ensure that the tools meet broader community requirements. The LabIMotion Hub plays a vital role in this process, serving as a central platform for the distribution and updating of components, thereby promoting standardization and continuous improvement across different ELN instances.

## Supplementary Information

Supplementary Material 1.

## Data Availability

The software component LabIMotion described in this work is available under an open source license from GitHub and Zenodo (https://github.com/LabIMotion/labimotion, https://doi.org/10.5281/zenodo.8305411 and https://zenodo.org/records/11062731). Official releases of LabIMotion begin with version 1.0.0, from which the full functionality outlined in the documentation (https://www.chemotion.net/docs/labimotion) has been fully integrated into Chemotion ELN version 1.8 and higher. Documentation and Installation: The requirements to install the Chemotion ELN are described in detail in the documentation of Chemotion ELN (https://www.chemotion.net/docs/eln/install_configure). For the work described here, a comprehensive documentation is available. The documentation covers information about the possible design of the components at different levels and summarizes the implemented methods and options for fields, layers and workflows (https://chemotion.net/docs/labimotion).Description of most important open-source components used for this work: React (https://react.dev/): The core library that is used for building the user interfaces (UIs). It is known for efficiently managing the presentation of components and supporting the development of interactive, state-driven web applications. React-Bootstrap (https://react-bootstrap.netlify.app/): A front-end framework that serves as a UI foundation for building consistent and responsive user interfaces. Ruby (https://www.ruby-lang.org/en/): A core language for back-end development. It is an open-source, dynamic, object-oriented programming language that powers web frameworks such as Rails. React Flow (https://reactflow.dev/): A library for building interactive graphs and visual workflows.

## Abbreviations

ELN: Electronic Lab Notebook
DB: Database
API: Application Programming Interface
FAIR: Findable, Accessible, Interoperable, Reusable
NFDI4Chem: National Research Data Infrastructure of Chemistry
GUI: Graphical User Interface
UI: User Interface
ACID: Atomicity, Consistency, Isolation, Durability
