# Supplementary material for: Addressing standardization and semantics in an electronic lab notebook for multidisciplinary use: LabIMotion
Source: J Cheminform. 2025 May 14;17:75. doi: 10.1186/s13321-025-01021-4 (PMC12080067; doi:10.1186/s13321-025-01021-4)
Supplement: Supplementary file 1 — Supplementary Material 1. [file 13321_2025_1021_MOESM1_ESM.pdf]

Addressing Standardization and Semantics in an Electronic Lab Notebook for Multidisciplinary Use: LabIMotion

Chia-Lin Lin[a], *Pei-Chi Huang*[a], *Christof Wöll*[b], *Patrick Théato*[c,d], *Christian Kübel*[e,f], *Lena Pilzb,*

*Nicole Jung**[a,f], *Stefan Bräse**[a,g]

Email: nicole.jung@kit.edu; stefan.braese@kit.edu

[a]Institute of Biological and Chemical Systems, Functional Molecular Systems (IBCS), Karlsruhe Institute of Technology, Kaiserstraße 12, 76131 Karlsruhe, Germany; [b]Institute of Functional Interfaces (IFG), Kaiserstraße 12, 76131 Karlsruhe, Germany; [c]Institute for Chemical Technology and Polymer Chemistry (ITCP), Karlsruhe Institute of Technology, Kaiserstraße 12, 76131 Karlsruhe, Germany; [d]Soft Matter Synthesis Laboratory, Institute for Biological Interfaces III (IBG3), Kaiserstraße 12, 76131 Karlsruhe, Germany; [e]Institute of Nanotechnology (INT), Karlsruhe Institute of Technology, Kaiserstraße 12, 76131 Karlsruhe, Germany; [f]Karlsruhe Nano Micro Facility (KNMFi), Kaiserstraße 12, 76131 Karlsruhe, Germany; [g]Institute of Organic Chemistry, Karlsruhe Institute of Technology, Kaiserstraße 12, 76131 Karlsruhe, Germany.

1. How to start **2**
2. Use Cases **5**
2.1 Extension for Polymer Chemistry 5
2.2 Reactions, samples and analyses for Metal organic frameworks 11
2.3 Workflows for TEM and SEM sample preparation 15
2.4 Discipline Agnostic Use Cases 17
2.5 Use case CO2-uptake of plants 18
3. Template Hub and distribution **20**
4. Supported fields **23**
3.1 Field types 23
3.2 Availability of the Supported fields in the available generic modules 26
5. Functions for the users **28**
4.1 Analysis Linkage for Elements 28
4.2 Workflow for Element 29
4.3 Dataset Metadata 30
4.4 Export and Import Collection 30
4.5 Search Elements 31
4.6 Element Counter 31
4.7 Revision for Element and Segment 32

1. How to start

When the Chemotion ELN standard modules do not meet the needs of a desired application and the ELN requires additional attributes, parameters, or workflows, scientists can use the LabIMotion extension. The adaptation of the core ELN can be done by two means (or the combination of both):

(A) The Template Hub provides several modules: If one of the available templates meets the requirements of a desired extension, the template can be simply synchronized to the ELN of the users.

(B) Scientists can create a template on their own. In this case, the invitation of experts in the same field to collaborate on the design is strongly recommended. Once the template is finalized, it can be released and accessed globally in the system.

Currently, there are three supported types of new modules:

- Generic Element: Elements are the highest level of information layer that can be created in the Chemotion ELN. The standard Elements available are "Reactions", "Samples", "Wellplates", "Screens" and "Research Plan". Elements usually consist of different Segments that describe their properties in detail. The most important Segment is "Properties", which usually contains the most important information on the Element. Generic Elements created with LabIMotion appear in the ELN UI as the standard Elements from Chemotion. Just the way how they are generated and how they are stored in the database differs as they are created with the LabIMotion extension. LabIMotion Generic Elements can be described as single entities with different Generic Segments attached. The LabIMotion generated Elements have additional capabilities as the standard Elements: They additionally support the generation of workflows.
- Generic Segment: Segments categorize more detailed information and relate it to the respective Element. While the standard Segments in Chemotion are usually "Properties", "Analyses", and "References" (some additional ones exist but are specific for a certain Element), the LabIMotion derived generic Segments can be created from scratch and can be assigned to any Element that's relevant.
- Generic Dataset: Datasets are automatically available when creating measurement data and selecting the corresponding ontology vocabulary. Generic Datasets have no counterpart in

Chemotion - they are a new option to generate Metadata descriptions for analytical data and can be applied to all analytical datasets that are stored in Chemotion.

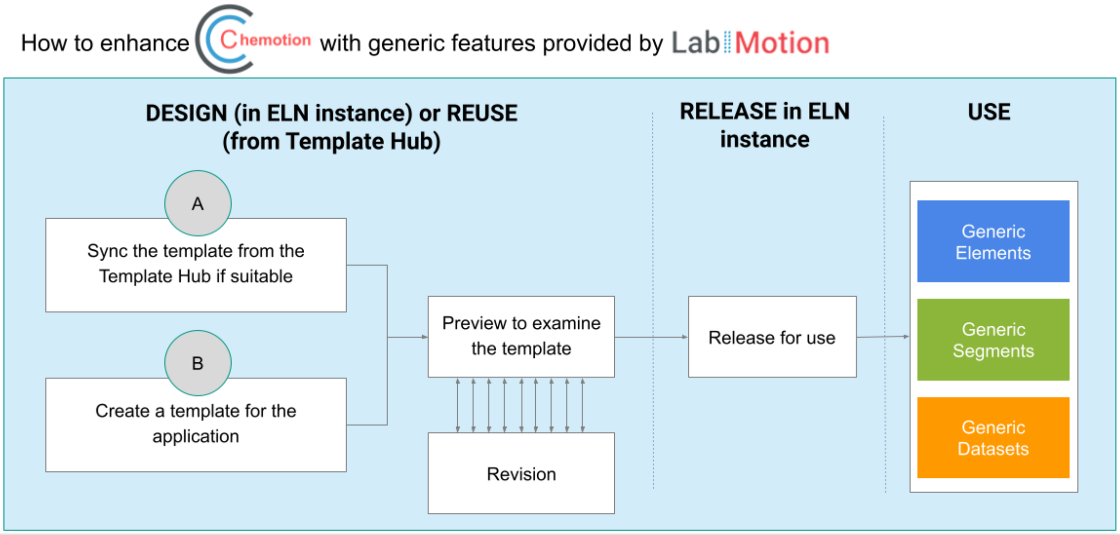

Figure S1. Workflow to use generic LabIMotion modules in Chemotion ELN: Alternative options A and B allow to either re-use existing extensions developed by others, or the design of new modules. Both options require the release of the selected modules in the used ELN instance - enabling the installation and use of three different types, either as a single extension or in combination of several extensions.

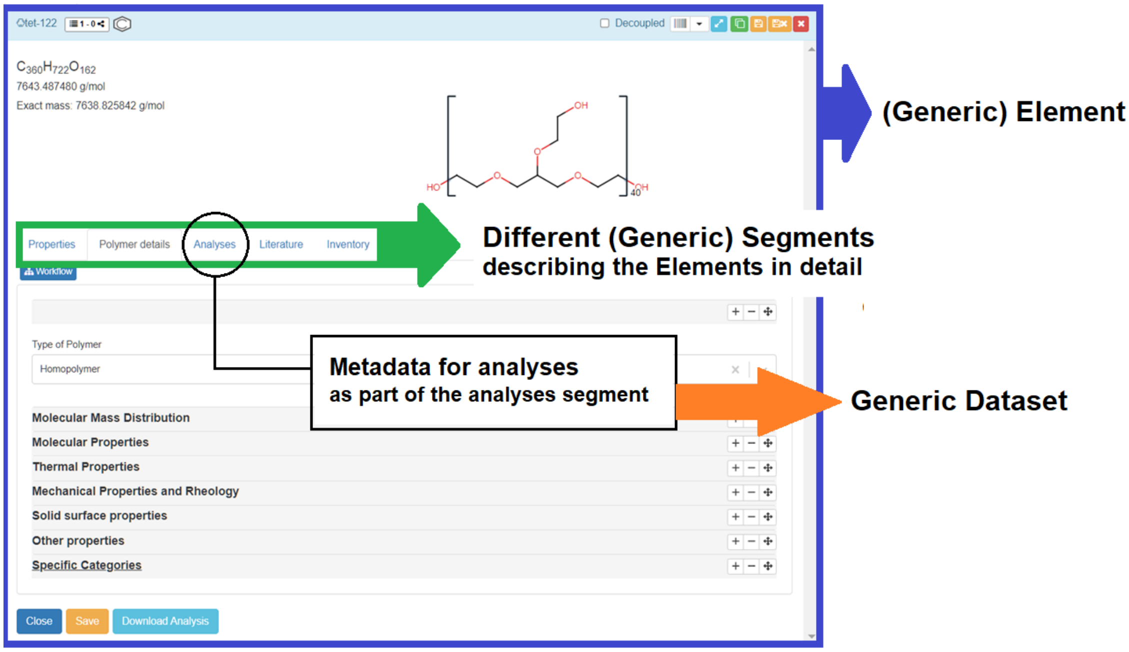

Figure S2. Explanation of the structure and relations of (Generic) Elements in blue, (Generic) Segments in green and Generic Datasets in orange as additional information to Figure 1 (main manuscript).

2. Use Cases

2.1 Extension for Polymer Chemistry

Challenge (from main manuscript): "Polymer chemistry as part of the domain of chemistry requires with respect to documentation (e.g. reaction) and analysis (e.g. nuclear magnetic resonance, NMR) a lot of functions that correspond to those of molecular chemists (e.g. organic and inorganic chemistry). Besides that, the polymer chemists need to record several characteristics in particular for polymer samples that are usually not described for standard chemistry samples. The analytical description of the sample requires additional analytical techniques such as thermogravimetric analysis (TGA), differential scanning calorimetry (DSC) and size exclusion chromatography (SEC)."

To cover the needs of this use case, we generated a *generic segment* (named *Polymer details*) (Figure S4) as an extension to the existing generic chemistry sample. This generic segment includes all relevant aspects to describe a polymer sample in detail, depending on the type of

polymer sample (Figure S3). In Figure S3 is demonstrated how the new segment is integrated with the already existing generic chemistry sample in Chemotion ELN, allowing to combine the aspects of the former Chemotion aspects with the new forms coming with the extension LabIMotion (*Polymer details*). Figure S4 showcases the layers of the new segment offering the documentation details according to different aspects of the sample description and Figure S5 gives details about the available input fields per layer. While the work on the extension of the sample description was enabled by the generation of a generic segment, the inclusion of new analysis types such as "TGA", "DSC" and "SEC" was achieved by the development of generic datasets (TGA shown in Figure S6). Also in the case of analytical data, LabIMotion is deeply integrated into the development of Chemotion (Figure S7). Figure S7 shows the analytical description for TGA, consisting of the core Chemotion elements providing different data file formats as representations of the original dataset by conversion of data into open file formats (Figure S7, left), and the combination of the data file area (called Attachments) with the metadata extracted from a file and represented with the generic datasets module of LabIMotion (Figure S7, right). The two areas "Attachments" and "Metadata" complement each other in the UI of Chemotion ELN.

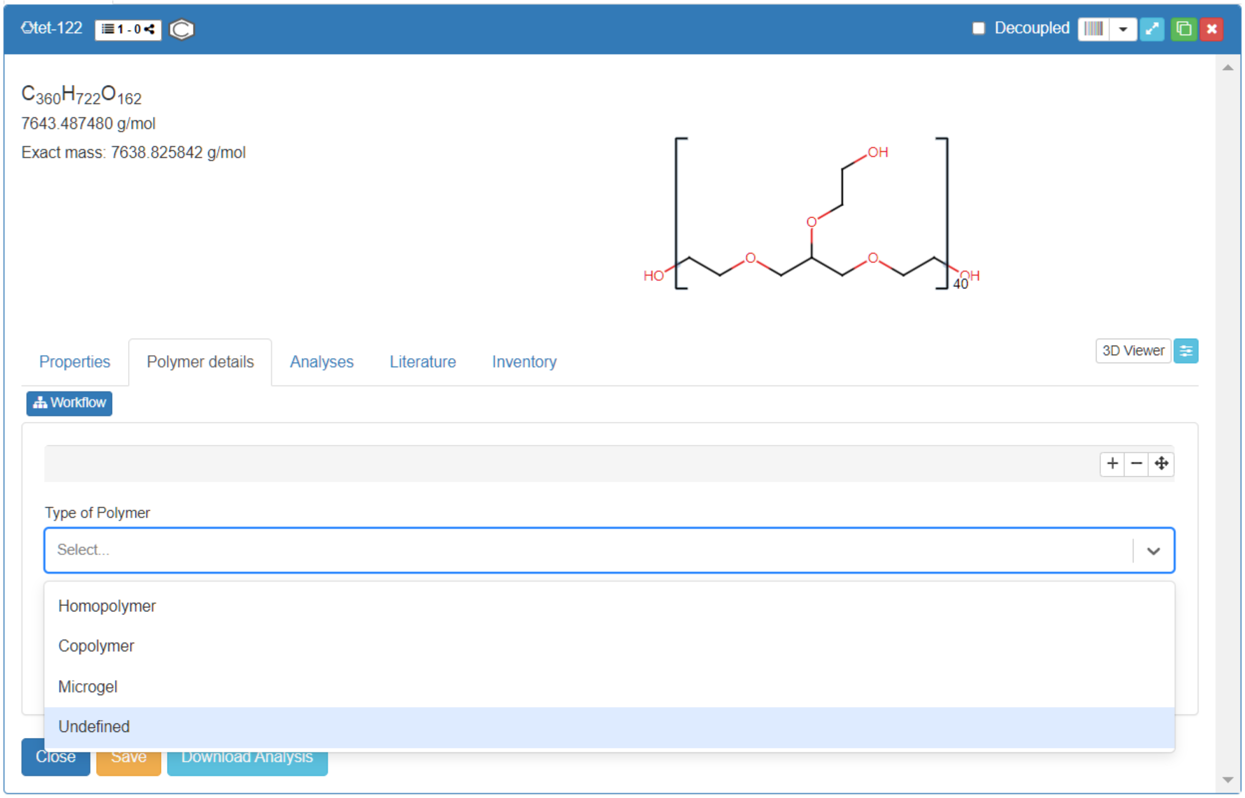

Figure S3. Screenshot of the way to choose the type of polymer extension to be given in the new segment polymer sample in Chemotion ELN with the extensions from LabIMotion. The UI of the ELN combines the former hard-coded general chemistry aspects such as drawing of the molecular structure and the general descriptions in the form of the *Properties* segment, *Analyses*, *Literature* and *Inventory*. The generic segment *Polymer details* which is part of the LabIMotion extension includes all relevant aspects to describe a polymer sample in detail (see Figure S5).

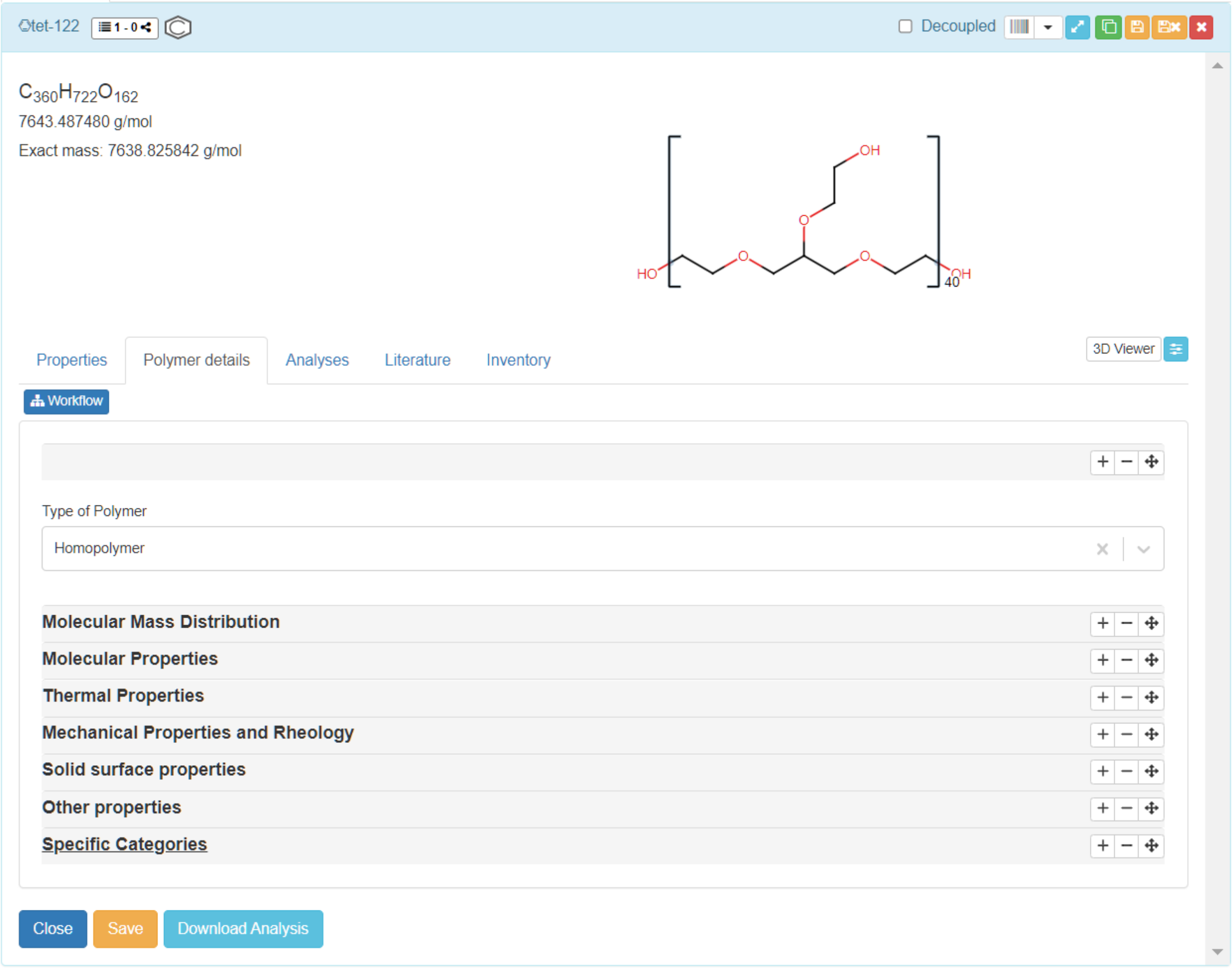

Figure S4. Screenshot of the options of the newly integrated generic segment *Polymer details* after the selection of the polymer type “Homopolymer”. All sections given in grey (seven sections from “Molecular Mass Distribution” to “Specific Categories”) can be opened and filled separately (see Figure S5). For the definition of the sections within this generic segment, we used different so-called layers within LabIMotion (main manuscript, Figure 4a).

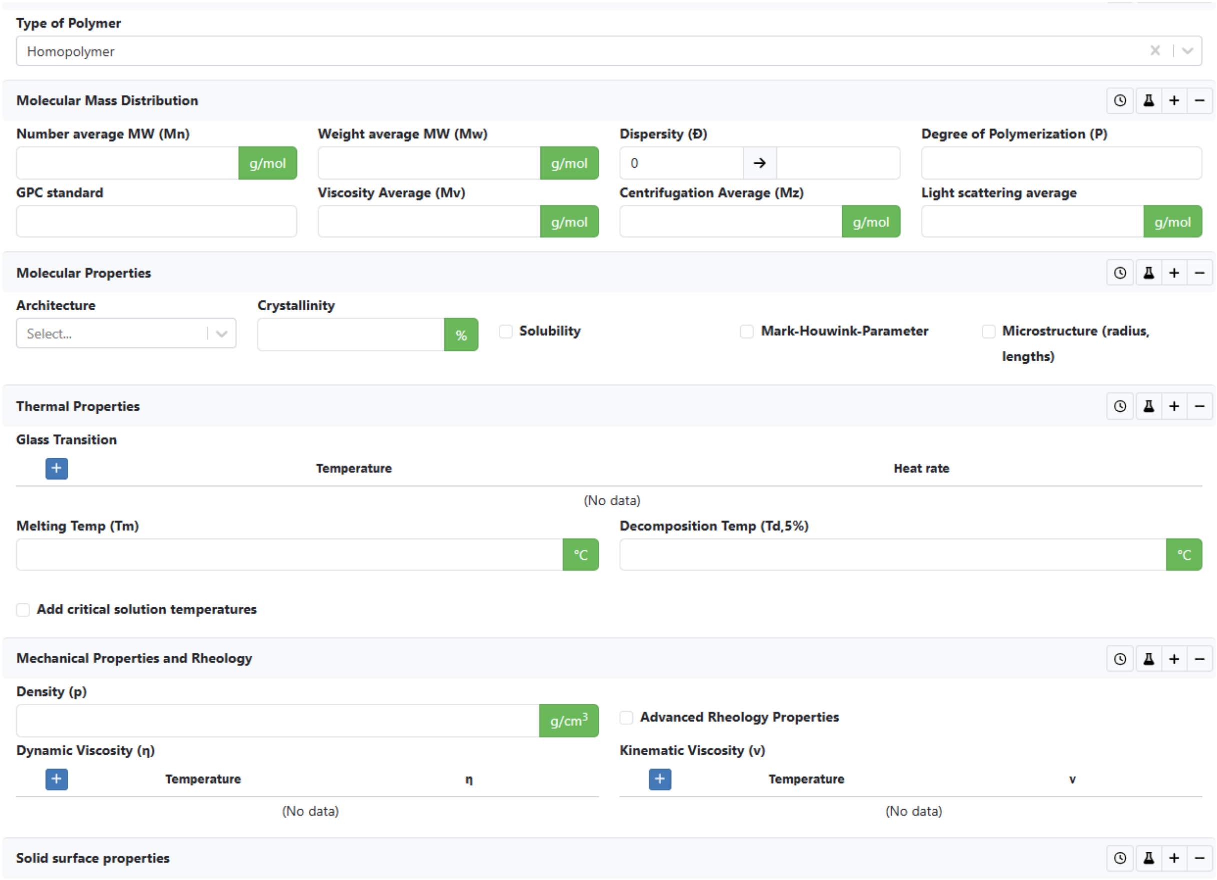

Figure S5. Screenshot showing the details available for the segment *Polymer details* after selection of the type "Homopolymer". Due to space reasons, only the details for the sections (technically called layers) "Molecular Mass Distribution", "Molecular Properties", "Thermal Properties" and "Mechanical Properties and Rheology" are shown. For giving the details in each layer, fields (main manuscript, Figure 4a) are used. Fields can be adapted to specific needs such as in the case of the Dispersity, which is a result of the calculation from Number average weight (Mn) and weight average (Mw). The system calculates the Dispersity with the input of the input fields Mn and Mw. The field function in that special case was "Text formula" (Table S2).

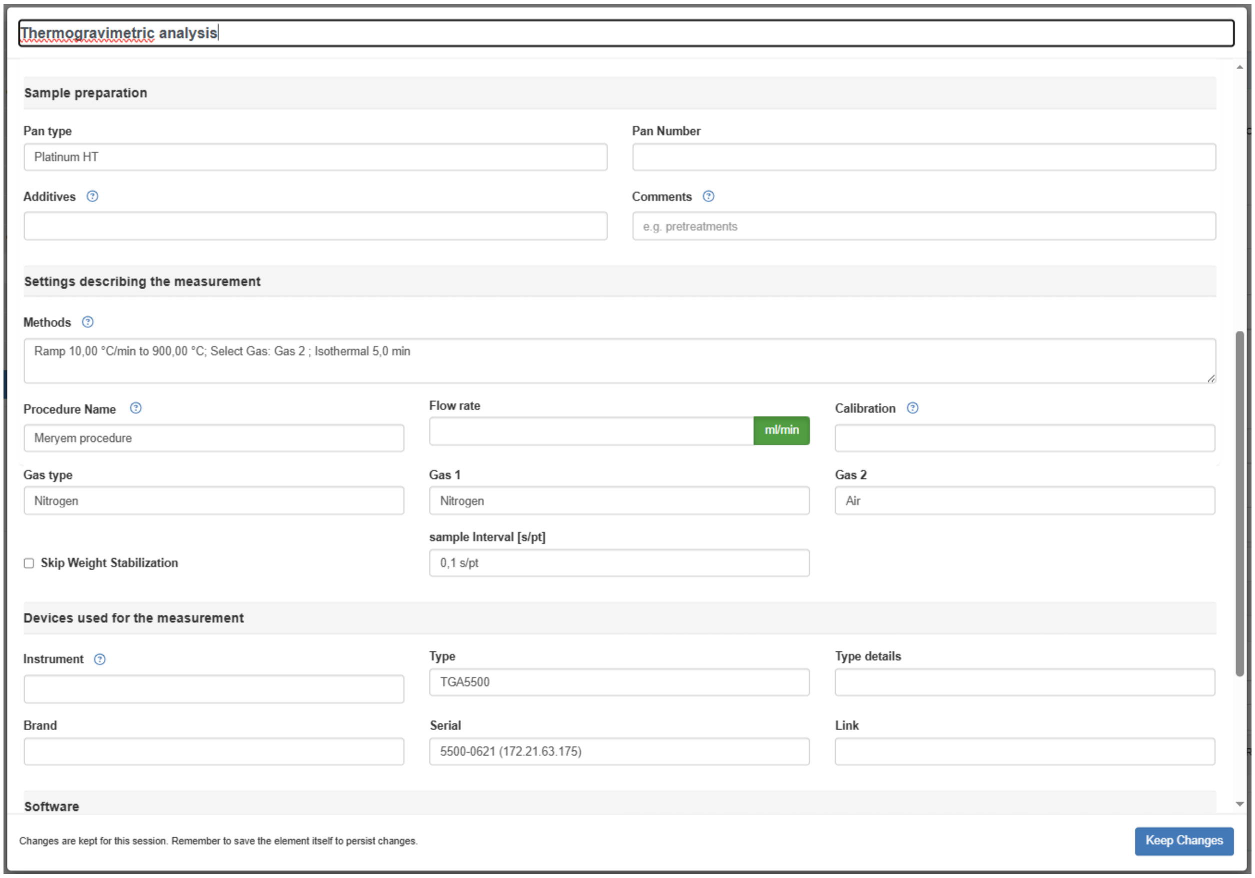

Figure S6. Details of the analysis method Thermogravimetric Analysis (TGA) represented with the LabIMotion extension allowing the design of *Generic Datasets*.

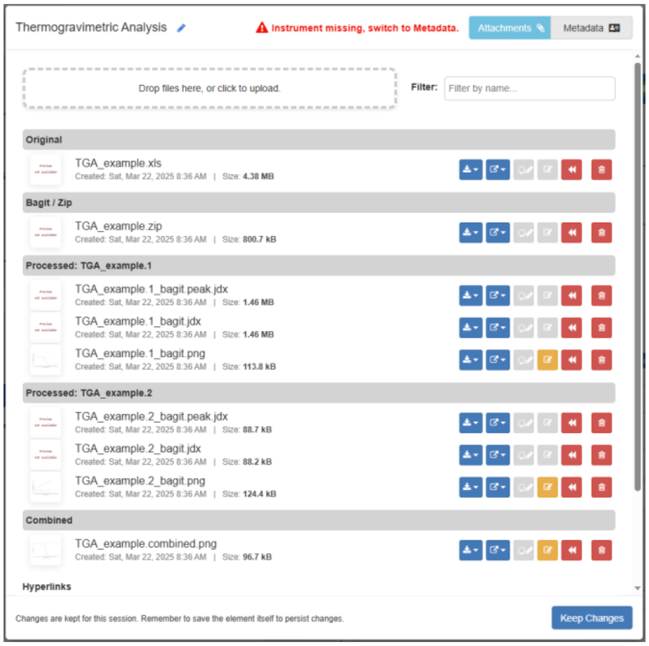

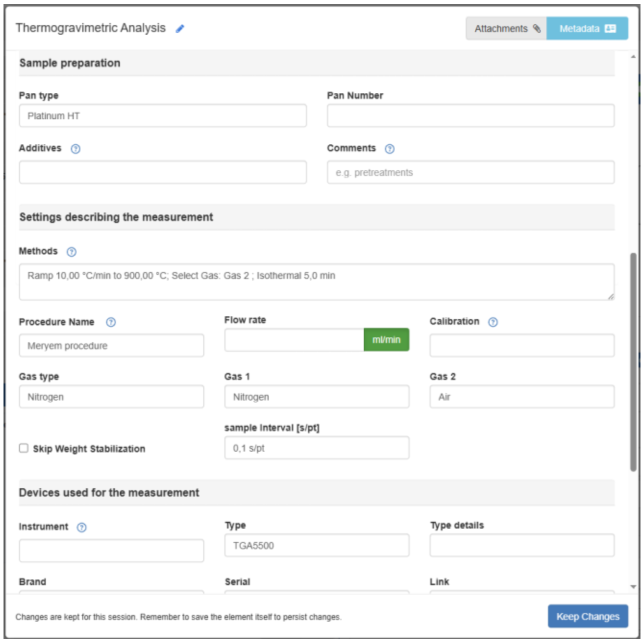

Figure S7. Screenshots showing the implementation of the *Generic Dataset* for Thermogravimetric Analysis (TGA) into the core functions of Chemotion ELN. Left: core functions of Chemotion giving different representations (different data file formats) of the original data file provided in the form of “Attachments”. Right: the UI switches to the “Metadata” of a TGA file, giving the metadata provided by the design of a *Generic Segment.* Both areas complement each other.

2.2 Reactions, samples and analyses for Metal organic frameworks

Challenge (from main manuscript): “Metal organic frameworks thematically bridge aspects of chemistry with materials sciences. Making an ELN suitable for science with MOFs, a suitable description of the preparation of MOFs and their description in the form of samples including analytical data is required. The analytical data section needs in particular an extension allowing the representation of single crystal X-ray diffraction (SCXD) and powder X-ray diffraction (PXRD). “

The challenges described for MOFs (including SURMOFs) were solved - similar to the solutions for polymer chemistry - by a combination of *Generic Segments* with *Generic Datasets*. To describe the synthesis of MOFs and SURMOFs in a suitable way, a *Generic Segment* was developed that reflects diverse methods to synthesize MOF materials. This segment was assigned to the already existing element “Reaction” of Chemotion ELN. The new segment extends the options of the former “Reaction” element (Figure S8) and is capable of describing MOFs as well as SURMOFs at the same time.

General setting for the synthesis of SURMOFs

Device	Type of sample holder	No of samples
TX-60	4-fold	2

Comments and further Details

Preparation of substrate

Substrate	Coating	
Silicon wafer	gold	

Form	Dimensions	Height of the substate
rectangle	1cm x 3cm	0.525 mm

Activation	Sam type	Activation: Duration
SAM	MHDA (16- Mercaptohexadecanoic acid)	72 h

Preparation of the used chemicals/materials in detail

Table 1: Listing of solvents and definition of solvent mixtures

Solvent label	Solvent A	Volume A	Solvent B	Volume B	Solvent C	Volume C	Ratio [A:B:C]
so...	Et...	210 ml	Select...	l	Select...	l	

Definition of synthesis cycle

Number of cycles

40

Table 3: Setting referring to materials and their conditions

Step number	Vessel number	Step type	Duration	Temperature [°C]
1	V1	metal installation	10 m	25
2	V2	spray cleaning	5 s	25
3	V3	sonication	58 s	25
4	V4	linker installation	15 m	25
5	V2	spray cleaning	5 s	25
6	V5	rinsing	5 m	25

Humidity [% rel.]	Atmosphere requirements	Other conditions
10 %	protective N2	

Termination type	Solvent of termination	Duration of termination step
drying		0 m

Comments and Observations

Fitness: 0,58

Figure S8. Screenshot of the description options for a SURMOF reaction. The layers (in grey) summarize different aspects of the synthesis in the form of an additional tab attached to the standard chemical synthesis area (called “Reaction”) in Chemotion ELN.

Additionally, a segment was developed as an extension to the general chemical sample, allowing to describe the characteristics of MOF materials in addition to the standard information provided for chemical samples (Figure S9).

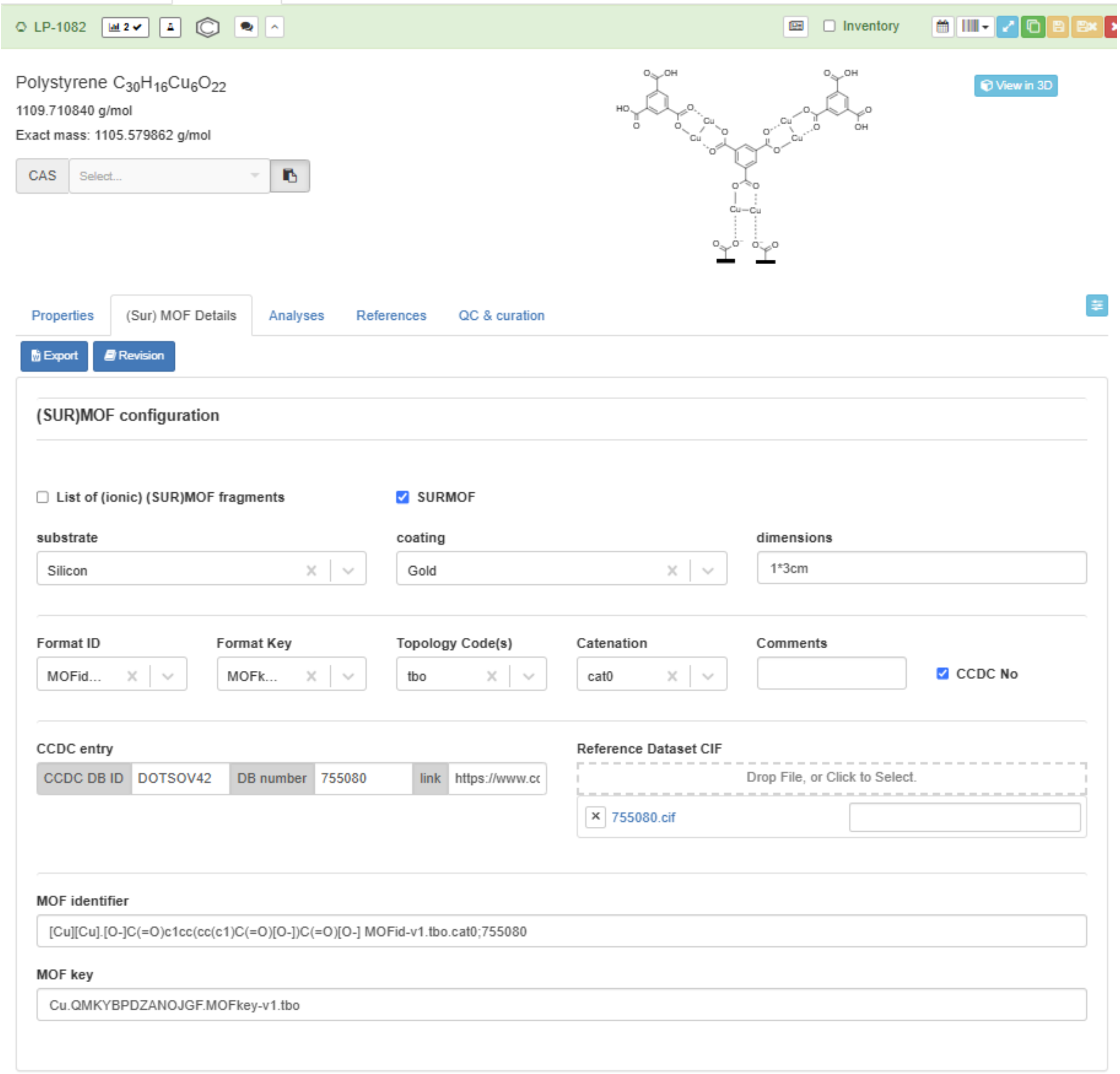

Figure S9. Segment for the definition of properties of a SURMOF material - provided as an extension to the Chemotion element “Sample”. The new segment *(SUR)MOF Details* summarizes

all characteristics of the material that are not part of the Chemotion descriptions “Properties”, “Analyses”, and “References”.

To meet the requirements in terms of analytical data representation, Generic Datasets were created for the techniques “Powder X-ray diffraction (XRD or PXRD)” and “Single Crystal X-ray diffraction (SCXD)”. In Figure 10, the integration of the additional Generic Dataset generated with the LabIMotion extension into the Chemotion ELN is depicted. While Chemotion offers the standard processing and visualization options for datasets which allows for representation of X-ray data e.g. as an image (Figure S10 top), the LabIMotion Generic Dataset enables the extraction and view of all metadata assigned to the analytical technique (Figure S10, bottom). Both parts are integrated into the UI thus that the user cannot identify which part comes from Chemotion and which part is a LabIMotion extension.

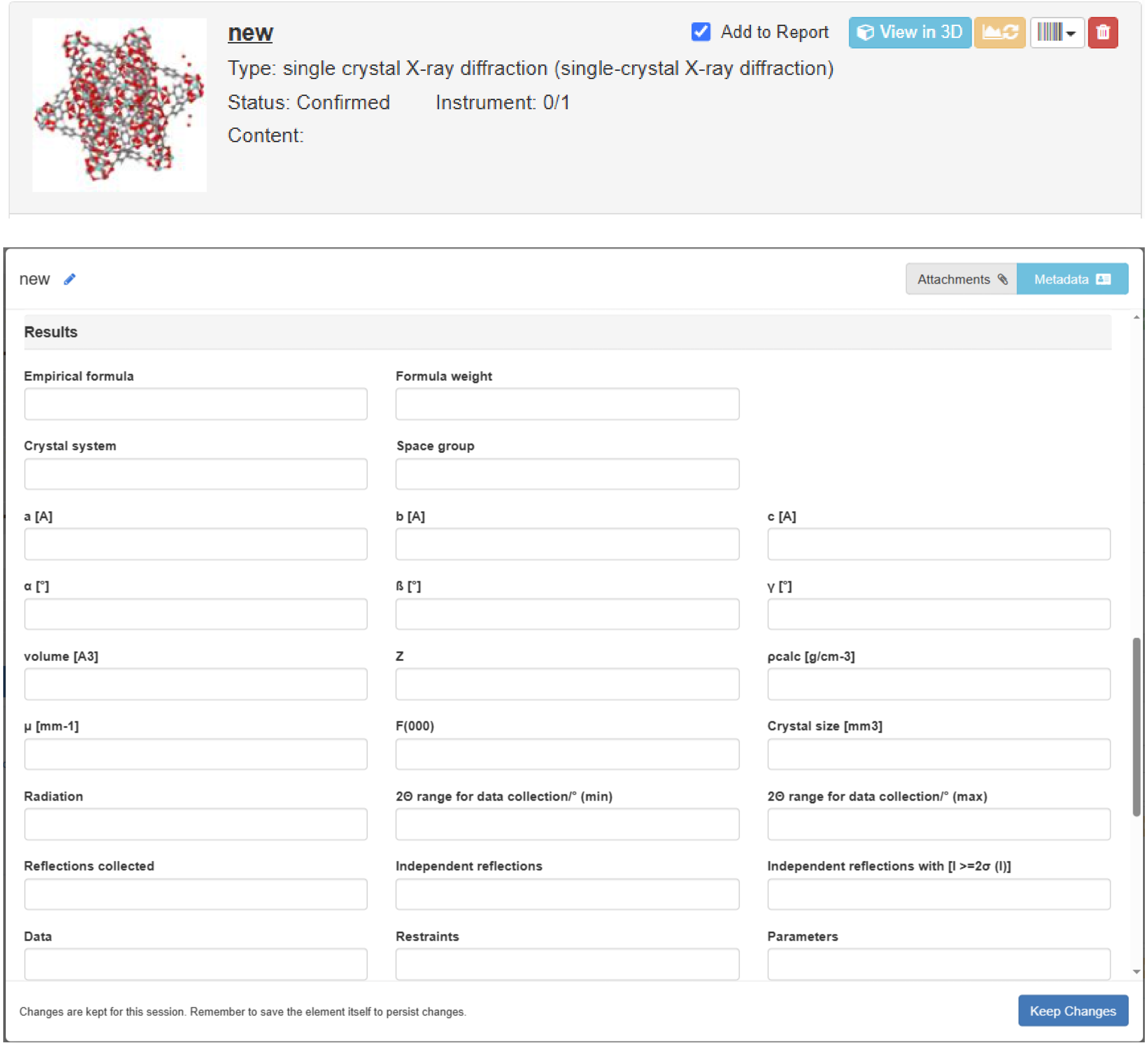

Figure S10. Screenshot of the representation of the technology SCXD in Chemotion ELN with the extension of LabIMotion. Upper part: the analysis section in Chemotion ELN where the SCXD data is visualized with an image of the crystal structure. Lower part: representation of the assigned metadata in the form of a Generic Dataset generated with LabIMotion.

2.3 Workflows for TEM and SEM sample preparation

Challenge (from main manuscript): "The description of TEM and SEM measurements which are used for the investigation of samples in many disciplines, require detailed documentation on the preparation of the samples in the form of a workflow containing details about different processing steps that are conducted with the sample."

The challenge was solved with the implementation of a *Generic Element* that consists of different Layers that can be placed in a workflow which allows the successive work on these layers in a step by step approach. Figure S11 visualizes the workflow as presented to the user (upper part) and the layers (with included fields) that correspond to each of the steps in the workflow (lower part).

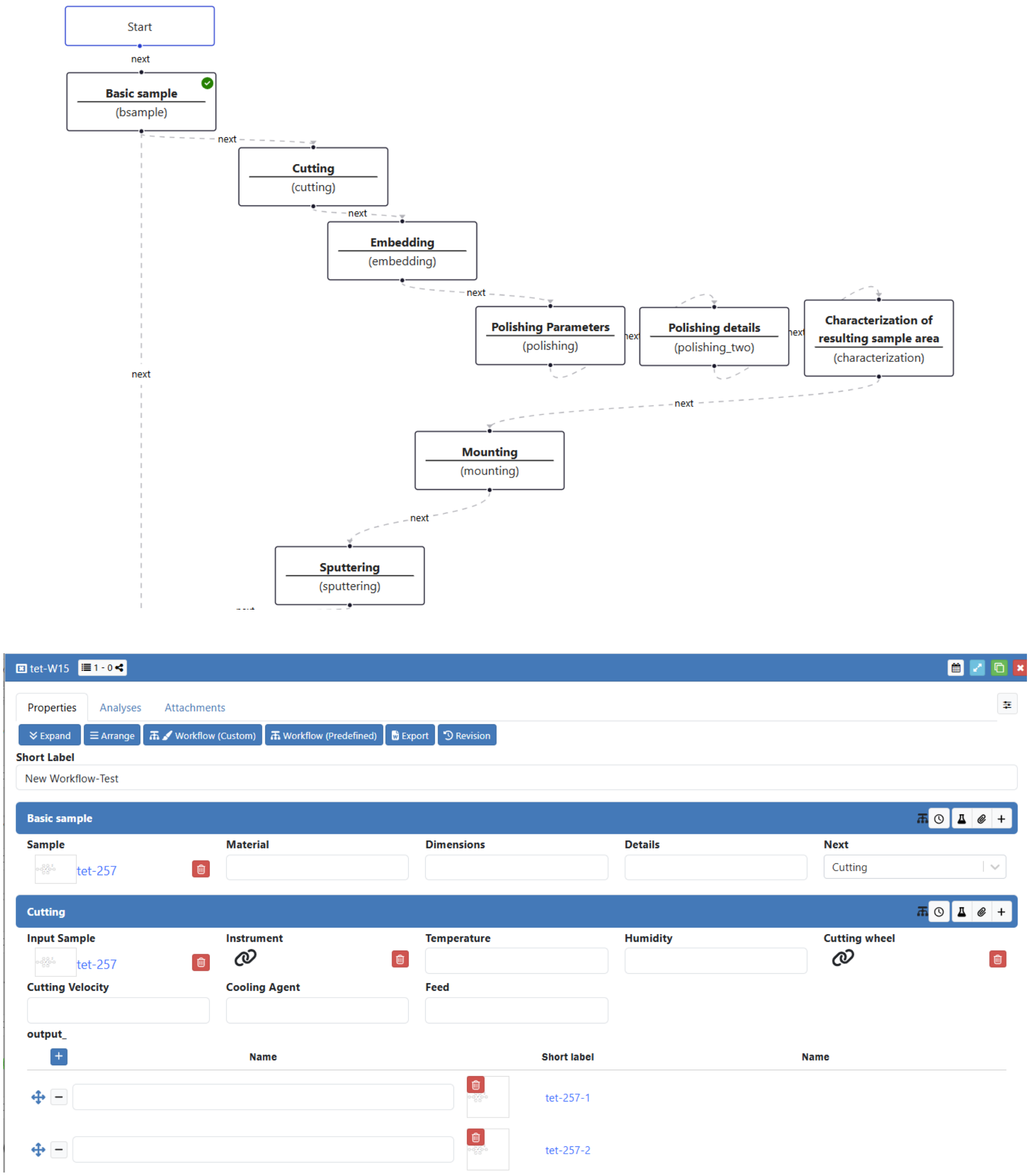

Figure S11. Screenshot showing the workflow used for sample preparations aiming to conduct TEM and SEM measurements. Upper part: workflow as presented to the user to give an overview about the overall progress of the documentation referring to all available layers, lower part: representation of selected layers (with included fields) that correspond to each of the steps in the workflow.

2.4 Discipline Agnostic Use Cases

To demonstrate the suitability of the LabIMotion extension for the documentation in disciplines that are not related to chemistry, a generic element designed to allow the documentation of devices/instruments is described. Devices (e.g. balances, analytical devices) are used for almost all scientific disciplines and their registration and description in an ELN allows for a standardized way to include the reference to a used device. The new *Generic element* "Device" includes all general metadata of the described device and can be either used alone (Figure S12) or in combination with an additional *Generic segment* to capture additional device specific information (not shown).

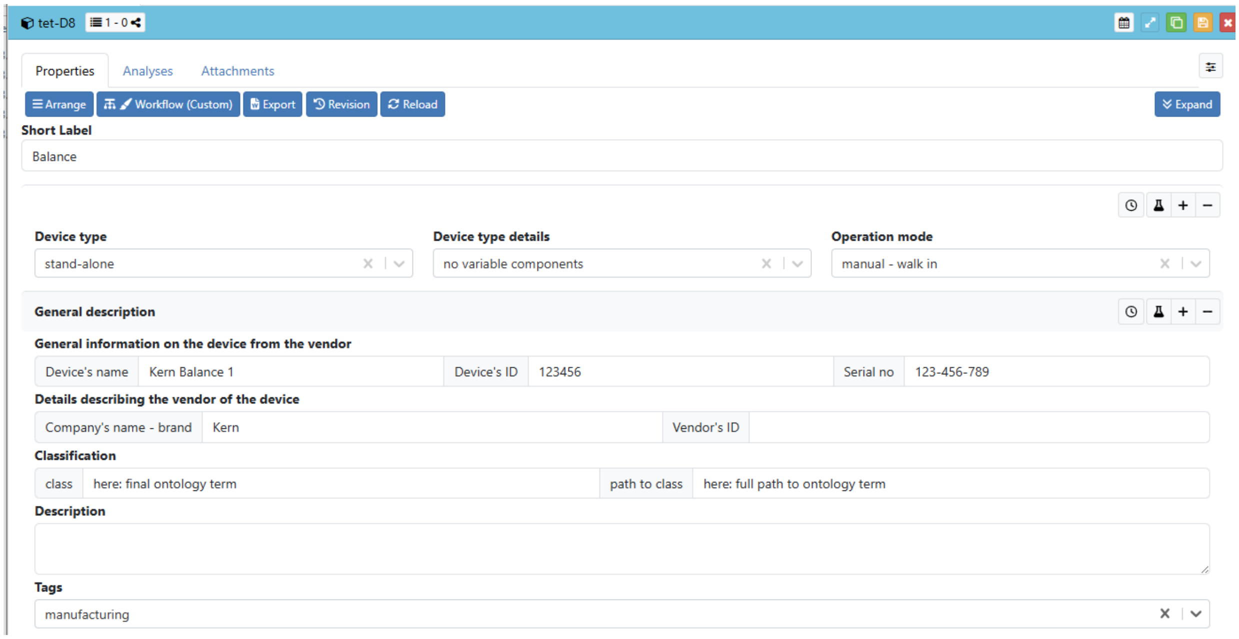

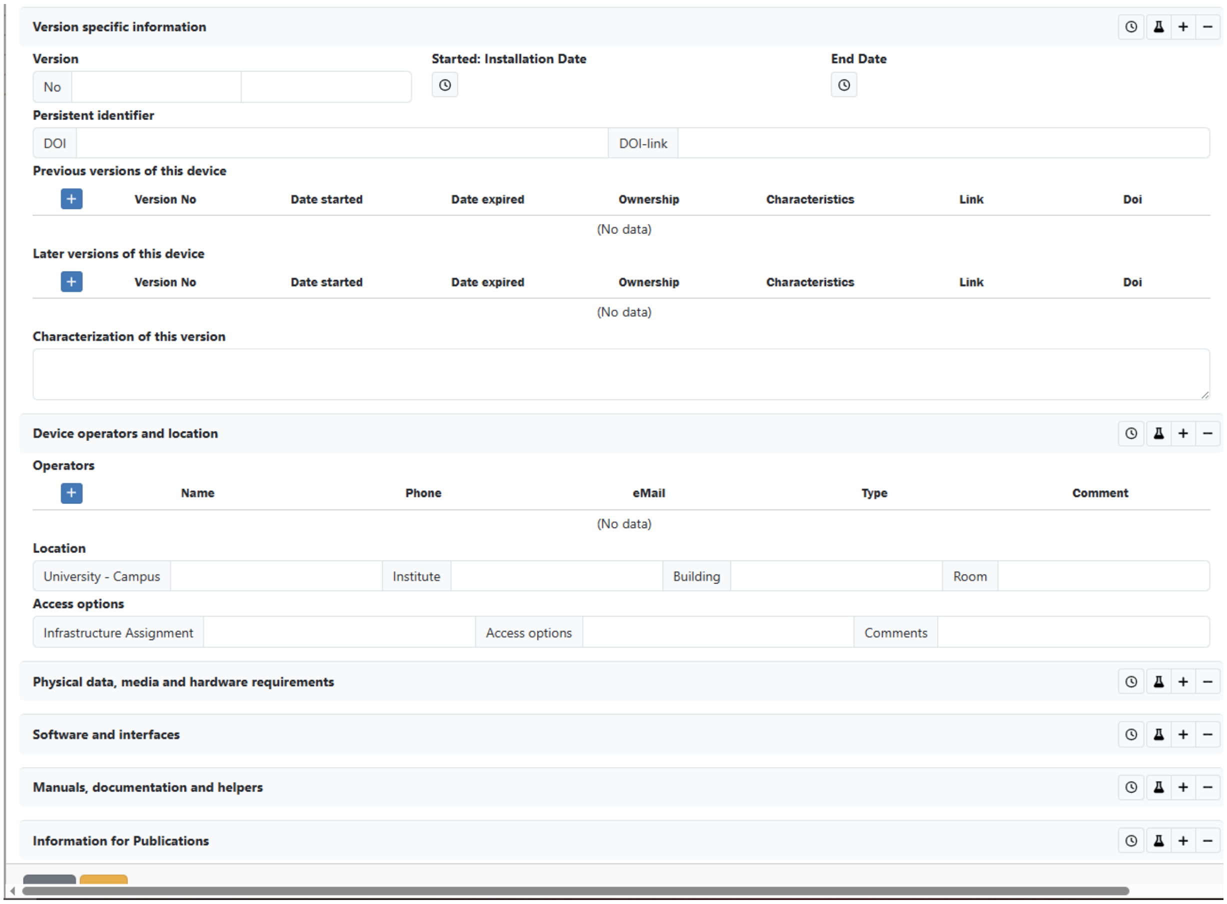

Figure S12. Screenshot taken from the device element designed with the LabIMotion extension of Chemotion ELN. Besides the categories included to the generic element (e.g. owners, identifiers, external links, versions available and others), analyses and attachments are available - allowing to assign device configurations, manuals or other information that is necessary to properly understand and use the device.

2.5 Use case CO2-uptake of plants

In order to demonstrate the use of the LabIMotion functions for plant biology applications, we selected an example of the past literature and mapped the experimental description given therein to a Generic Element. Figure S13 showcases how the work done by others in the past[1] could be integrated into an ELN-environment that offers a flexible approach referring to the integration of new descriptions (e.g. the combination of Layers and Fields). In particular the ability to add information in the form of predefined value-unit blocks via the LabIMotion field type “system-defined field” allowed a straightforward implementation of the work. The example also shows

where LabIMotion still needs extensions: e.g. some units (photon flux density, µmol/m^{2}s) are not included yet and need to be added to the portfolio.

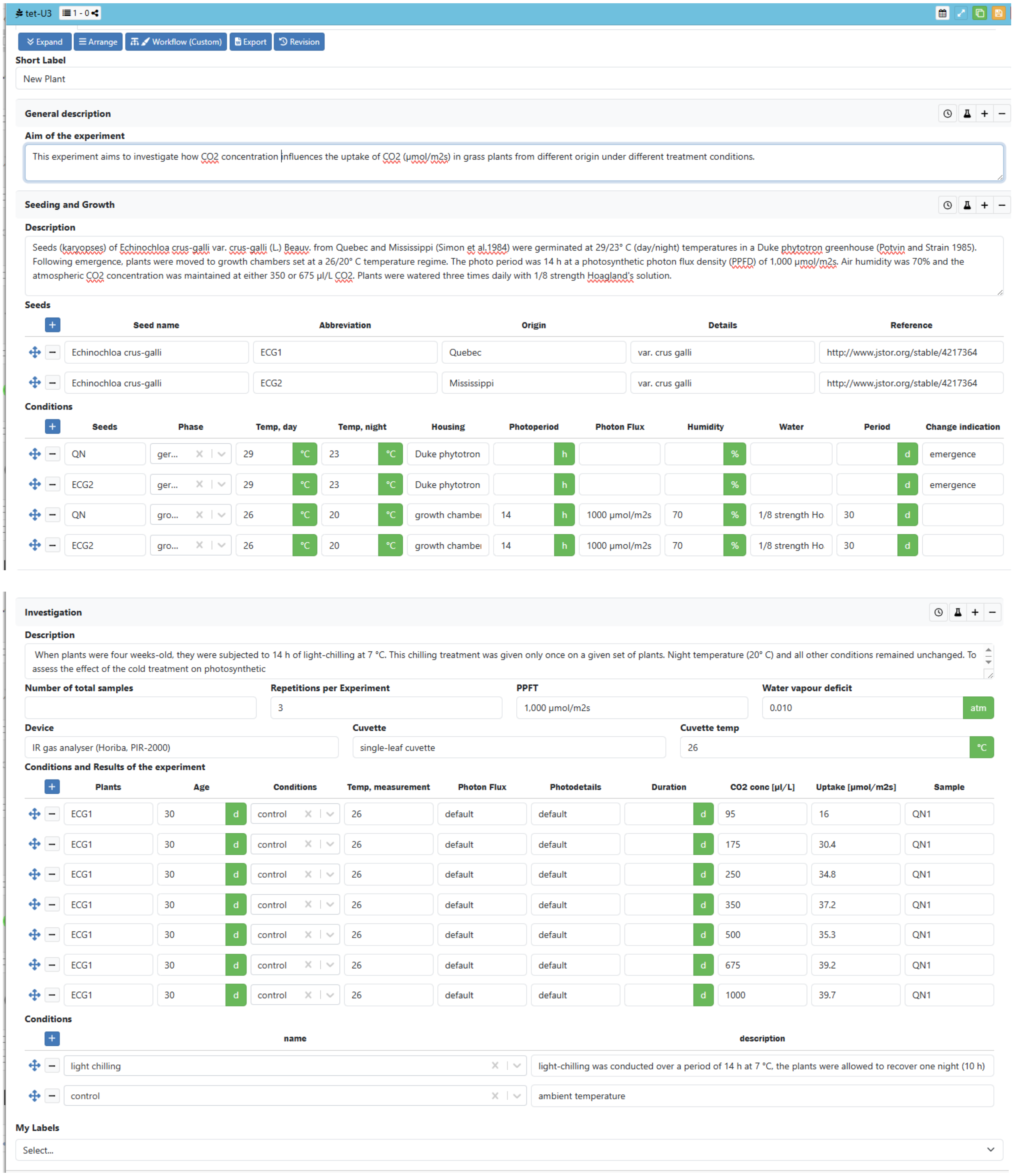
tet-U3 1 - 0

Expand | Arrange | Workflow (Custom) | Export | Revision

Short Label

New Plant

General description

Aim of the experiment

This experiment aims to investigate how CO2 concentration influences the uptake of CO2 (µmol/m2s) in grass plants from different origin under different treatment conditions.

Seeding and Growth

Description

Seeds (karyopses) of Echinochloa crus-galli var. crus-galli (L.) Beauv. from Quebec and Mississippi (Simon et al.1984) were germinated at 29/23° C (day/night) temperatures in a Duke phytotron greenhouse (Potvin and Strain 1985). Following emergence, plants were moved to growth chambers set at a 26/20° C temperature regime. The photo period was 14 h at a photosynthetic photon flux density (PPFD) of 1,000 µmol/m2s. Air humidity was 70% and the atmospheric CO2 concentration was maintained at either 350 or 675 µl/L CO2. Plants were watered three times daily with 1/8 strength Hoagland's solution.

Seeds

Seed name	Abbreviation	Origin	Details	Reference
Echinochloa crus-galli	ECG1	Quebec	var. crus galli	http://www.jstor.org/stable/4217364
Echinochloa crus-galli	ECG2	Mississippi	var. crus galli	http://www.jstor.org/stable/4217364

Conditions

Seeds	Phase	Temp, day	Temp, night	Housing	Photoperiod	Photon Flux	Humidity	Water	Period	Change indication
QN	ger...	29 °C	23 °C	Duke phytotron	h		%		d	emergence
ECG2	ger...	29 °C	23 °C	Duke phytotron	h		%		d	emergence
QN	gro...	26 °C	20 °C	growth chambe	14 h	1000 µmol/m2s	70 %	1/8 strength Ho	30 d	
ECG2	gro...	26 °C	20 °C	growth chambe	14 h	1000 µmol/m2s	70 %	1/8 strength Ho	30 d	

Investigation

Description

When plants were four weeks-old, they were subjected to 14 h of light-chilling at 7 °C. This chilling treatment was given only once on a given set of plants. Night temperature (20° C) and all other conditions remained unchanged. To assess the effect of the cold treatment on photosynthetic

Number of total samples | Repetitions per Experiment: 3 | PPFT: 1,000 µmol/m2s | Water vapour deficit: 0.010 atm

Device: IR gas analyser (Horiba, PIR-2000) | Cuvette: single-leaf cuvette | Cuvette temp: 26 °C

Conditions and Results of the experiment

Plants	Age	Conditions	Temp, measurement	Photon Flux	Photodetails	Duration	CO2 conc [µl/L]	Uptake [µmol/m2s]	Sample
ECG1	30 d	control	26	default	default	d	95	16	QN1
ECG1	30 d	control	26	default	default	d	175	30.4	QN1
ECG1	30 d	control	26	default	default	d	250	34.8	QN1
ECG1	30 d	control	26	default	default	d	350	37.2	QN1
ECG1	30 d	control	26	default	default	d	500	35.3	QN1
ECG1	30 d	control	26	default	default	d	675	39.2	QN1
ECG1	30 d	control	26	default	default	d	1000	39.7	QN1

Conditions

name	description
light chilling	light-chilling was conducted over a period of 14 h at 7 °C, the plants were allowed to recover one night (10 h)
control	ambient temperature

My Labels

Select...

Figure S13. Use of the LabIMotion functions to generate a *Generic Element* for recording information on the CO2 uptake of plants under different conditions. The entry was created based on published work of others from 1986.[1,2]

Use Case	Challenge	Generic Element	Generic Segment*	Generic Dataset
Polymer Chemistry	Polymer Sample		x (Sample)	
	Analytics (TGA, DSC, SEC)			x
MOF/SURMOF	Synthesis description		x (Reaction)	
	Sample description		x (Sample)	
	Analytics (XRD, SCXD)			x
TEM/SEM sample preparation	workflow required	x		
Device (Discipline Agnostic)	new element needed	x	(x) (Device)	
CO2 uptake	new process needed	x		

Table S1. Summary of the presented use-case and the means by which the challenges of the use cases were solved. *for *Generic Segment* brackets indicate to which *Element* the extension in the form of the *Generic Segment* is assigned.

3. Template Hub and distribution

As a designer, one has the ability to request templates for incorporation into the instance. Once these templates are requested (from the Template Hub), the templates will be generated and subsequently made available within the instance, allowing users to promptly start their utilization.

If the designer has synchronized a template and a new version is available in the LabIMotion Template Hub, synchronizing the template again will update it to the latest version. The designer has the option to preview the template or customize the design if necessary.

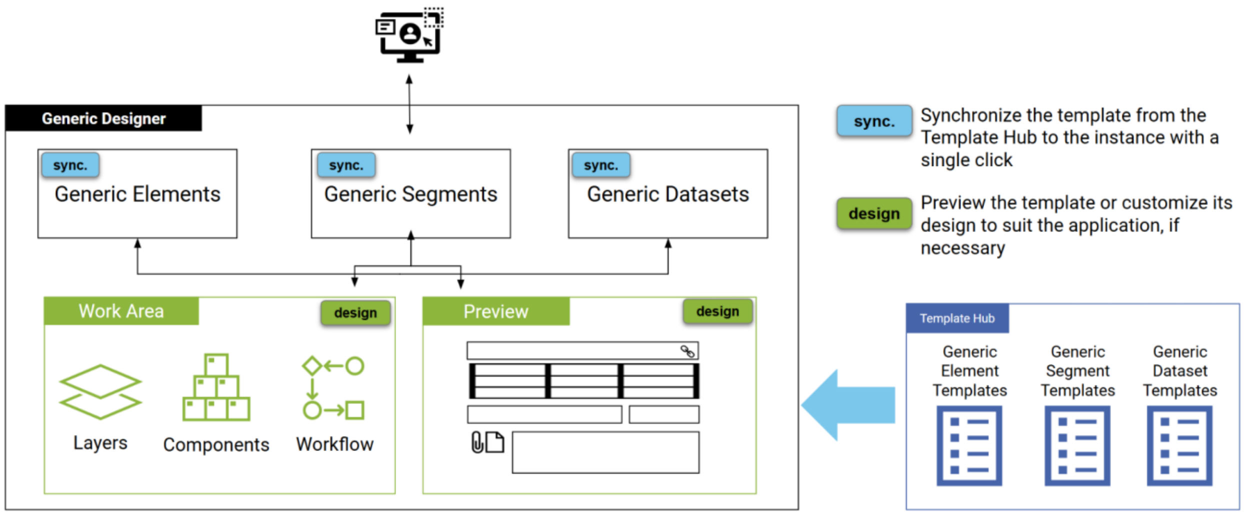

Figure S14. Summary of the sync and design options for the Generic Modules.

The released template includes a version number, following the format **(major).(minor)**. When the designer releases it for the first time, it is assigned an initial version number (major release: 1.0, minor release: 0.1). Users can only access the template after it has been released by the designer.

- If the designer clicks "**Save and Release (Major)**", the version number changes to the next major version. For example, if the current version is 2.5, the new version after release will be 3.0.
- If the designer clicks "**Save and Release (Minor)**", the version number increases by one minor version. For instance, if the current version is 2.5, the next version will be 2.6.

When the designer synchronizes a template from the LabIMotion Template Hub, the version number of the template is also being synchronized. After synchronizing from the Template Hub, the initial state of the template is inactive. The designer needs to activate it to make it available for users. However, if no changes are required, the designer does not need to release the template again to create a new version number, so that the version number stays the same as the version number in LabIMotion Template Hub.

Chemical Methods Ontology	Version	Released at
gas chromatography-mass spectrometry (GCMS)	0.1	2023-10-23T09:31:27.894Z
single crystal X-ray diffraction (single-crystal X-ray diffraction)	0.1	2023-11-15T15:05:30.992Z
size-exclusion chromatography (SEC)	0.1	2023-09-07T11:35:21.578Z
sorption-desorption measurement(sorption-desorption)	0.2	2023-11-02T12:30:47.856Z
gas chromatography (GC)	0.1	2023-11-20T13:42:59.155Z
X-ray diffraction (XRD)	0.1	2023-12-21T10:52:45.126Z
13C nuclear magnetic resonance spectroscopy (13C NMR)	1.2	2024-07-23T12:48:42.858Z
cyclic voltammetry (CV)	0.5	2024-08-26T09:25:19.081Z
ultraviolet-visible spectrophotometry (UV-VIS)	0.1	2024-05-02T07:27:29.536Z
1H nuclear magnetic resonance spectroscopy (1H NMR)	0.2	2024-07-23T12:47:38.189Z

Figure S15. Exemplary view on generic datasets within a selected ELN instance from the viewpoint of a designer.

The **Revision** feature displays the 10 most recent versions of the released templates, including their version UUIDs and release times in UTC format. Each version supports the following actions: **Preview**, **Retrieve**, **Download**, and **Delete**.

Preview This Version: The preview feature is available for all versions, including the current version. This allows examination of the template content before taking further actions.

Retrieve: Each version of the template can be retrieved. Once retrieved, the version will be set to draft status. Releasing the version is required before it can be used.

Download: Each version of the template can be downloaded as a `*.json` file. The file name following the naming convention includes the system prefix, template name, and version UUID, separated by an underscore(_). For example, if the system prefix is `abc`, the template name is `template123`, and the version UUID is `9b0c302c-6755-49bf-aea9-92099213c3dc`, the file name will be `abc_template1-9b0c302c-6755-49bf-aea9-92099213c3dc.json`.

Delete: Old versions can be deleted if they are no longer needed. This action is irreversible and cannot be undone once completed.

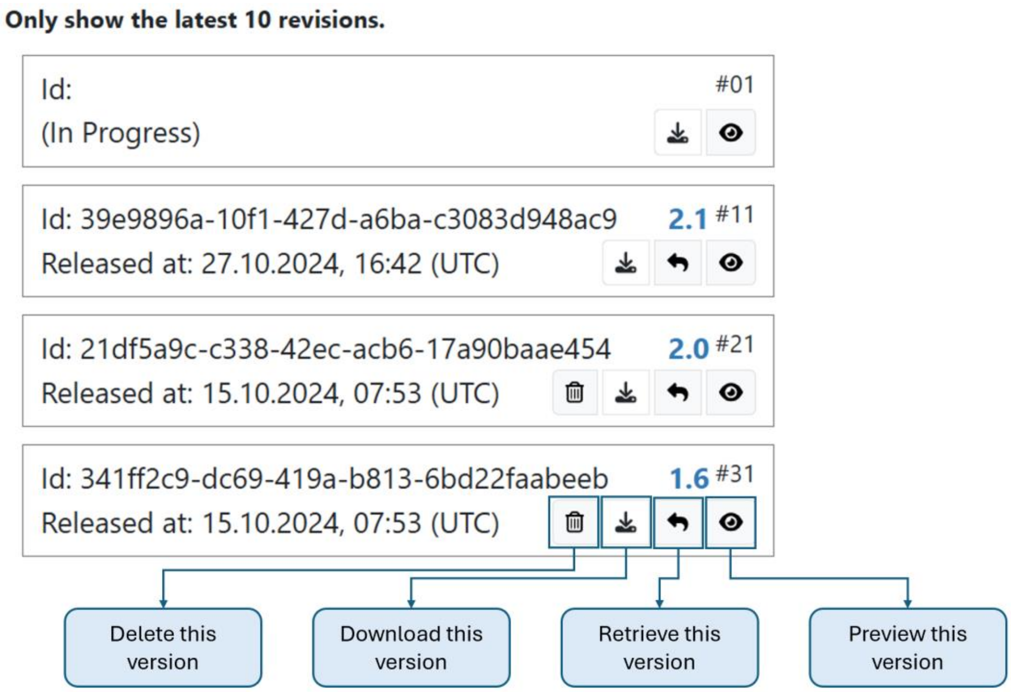

Figure S16. Screenshot of the list of versions within the designer view, including the basic functions delete, download, retrieve and preview available for each version.

4. Supported fields

4.1 Field types

4.1.1 Checkbox

The checkbox type field is used to capture a binary choice, typically representing a true/false, on/off or yes/no decision. It appears as a square box in user view with a tick or untick option, and the default value of the checkbox is unticked (false/off/no). It can be used as a restrictive field. Therefore, the restriction value must be entered as either "yes" or "no" (alternatively, "1" or "0").

4.1.2 Date-Time Picker

The Date-Time Picker type field allows users to select or enter a specific date and time from a calendar or input field. It's shown as a clock icon if it's empty in the user view, otherwise it displays the date time with format "DD/MM/YYYY hh:mm".

4.1.3 Datetime Range

The Datetime Range type field enables users to define a range between two specific dates and times. Enter a starting time (Start) by simply clicking the clock-button at the first section or enter a date time manually. By clicking the clock-button in the next section (Stop) a time interval (Duration) will be calculated. Users can switch between seconds (sec(s)), minutes (min(s)), hours (hour(s)), and days (day(s)) and the system will do the calculation. The Datetime Range field always takes up a full line in the layout.

4.1.4 Drag Element

The Drag Element type field features an icon with drag-and-drop functionality, allowing users to seamlessly embed a generic element by simply dragging and dropping it. Upon adding, the icon of the element is displayed for easy reference.

4.1.5 Drag Molecule

The Drag Molecule type field features an icon with drag-and-drop functionality, allowing users to seamlessly embed a molecule by simply dragging and dropping it. Upon adding, the molecule image and its name is displayed for easy reference.

4.1.6 Drag Sample

The Drag Sample type field features an icon with drag-and-drop functionality, allowing users to seamlessly embed a sample by simply dragging and dropping it. Upon adding, the sample image is displayed for easy reference. An icon with drag & drop function to let the user add a sample. It will display the sample image after being added.

4.1.7 Formula-Field

The Formula-Field type field is used to perform calculations based on specified inputs. Incorporate other fields by simply using their unique Field Name and mathematical operations like addition (+), subtraction (-), multiplication (*), and division (/). These mathematical operations are executed automatically, and the output decimals can be limited if the user specifies the number of decimals. The result of the calculation can also be adjusted manually by the user.

4.1.8 Input Group

The Input Group field type combines multiple fields and displays them as a single field. It allows combining labels, text, numbers and System-Defined fields to efficiently extend the input functionality.

4.1.9 Integer

The Integer type field is used for storing numbers without decimals.

4.1.10 Select

The Select type field offers users a dropdown menu with a curated list of predefined options. Users can conveniently choose from this list, which consists of custom-defined values.

4.1.11 System-Defined

The System-Defined type field consists of a numeric field and a corresponding unit selector. This allows users to input a value and select from a predefined set of units. For example, if the unit selector is set to "Temperature" with the default unit as Celsius (°C), entering 100 in the input field and switching the unit to Fahrenheit (°F) will display the converted value 212 in the input field. The default units can be set, and the result of the calculation is rounded to 5 decimal places.

4.1.12 Table

The Table type field presents structured data in a tabular format, allowing users to view, edit, and manage multiple records. Various field types can be used in tables, including Drag Molecule, Drag Sample (Generic Element only), Select, System-Defined, and Text. For the Drag Sample field type, options are available to display additional information such as its Name, External Label, and Mass. Similarly, for the Drag Molecule field type, the available options include InChiKey, SMILES, IUPAC, and Mass.

4.1.13 Text

The Text type field is used for entering and displaying textual information. It can be used to enter any short-written information.

4.1.14 Text-Formula

The Text-Formula type field provides a dynamic text output that combines multiple text fields from the same layer and other layers in one display. This field type is useful when users want to display a text that is a combination of several text fields. The Text fields can be separated by different separators like space, comma, etc. The default separator is a dot (.).

4.1.15 Textarea

A Textarea type field provides a larger input area for users to enter and edit multi-line textual content.

4.1.16 Upload

The Upload type field is used for uploading and storing files. A file can be uploaded via drag & drop or browsing the computer.

4.1.17 Dummy

The Dummy field serves as a placeholder to occupy space within a column and remains invisible to users.

4.2 Availability of the Supported fields in the available generic modules

Supported fields	number	Element	Segment	Dataset
Checkbox	3.1	Yes	Yes	Yes
Date-Time Picker	3.2	Yes	Yes	Yes
Datetime Range	3.3	Yes	Yes	Yes
Drag Element	3.4	Yes	Yes	No
Drag Molecule	3.5	Yes	Yes	No
Drag Sample	3.6	Yes	Yes	No

Formula-Field	3.7	Yes	Yes	Yes
Input Group	3.8	Yes	Yes	No
Integer	3.9	Yes	Yes	Yes
Select	3.10	Yes	Yes	Yes
System defined	3.11	Yes	Yes	Yes
Table	3.12	Yes	Yes	No
Text	3.13	Yes	Yes	Yes
Text Formula	3.14	Yes	Yes	No
Text Area	3.15	Yes	Yes	Yes
Upload	3.16	Yes	Yes	No
Dummy field	3.17	Yes	Yes	Yes

Table S2. Summary of the supported field types in LabIMotion and their availability in the different modules generic element, generic segment and generic dataset.

5. Functions for the users

5.1 Analysis Linkage for Elements

The Analysis Linking feature allows users to embed Element's analyses into Element's workspace (Properties). With little effort, users can create links between different analysis processes, so they can view, mark or adjust related analysis data in the same workspace.

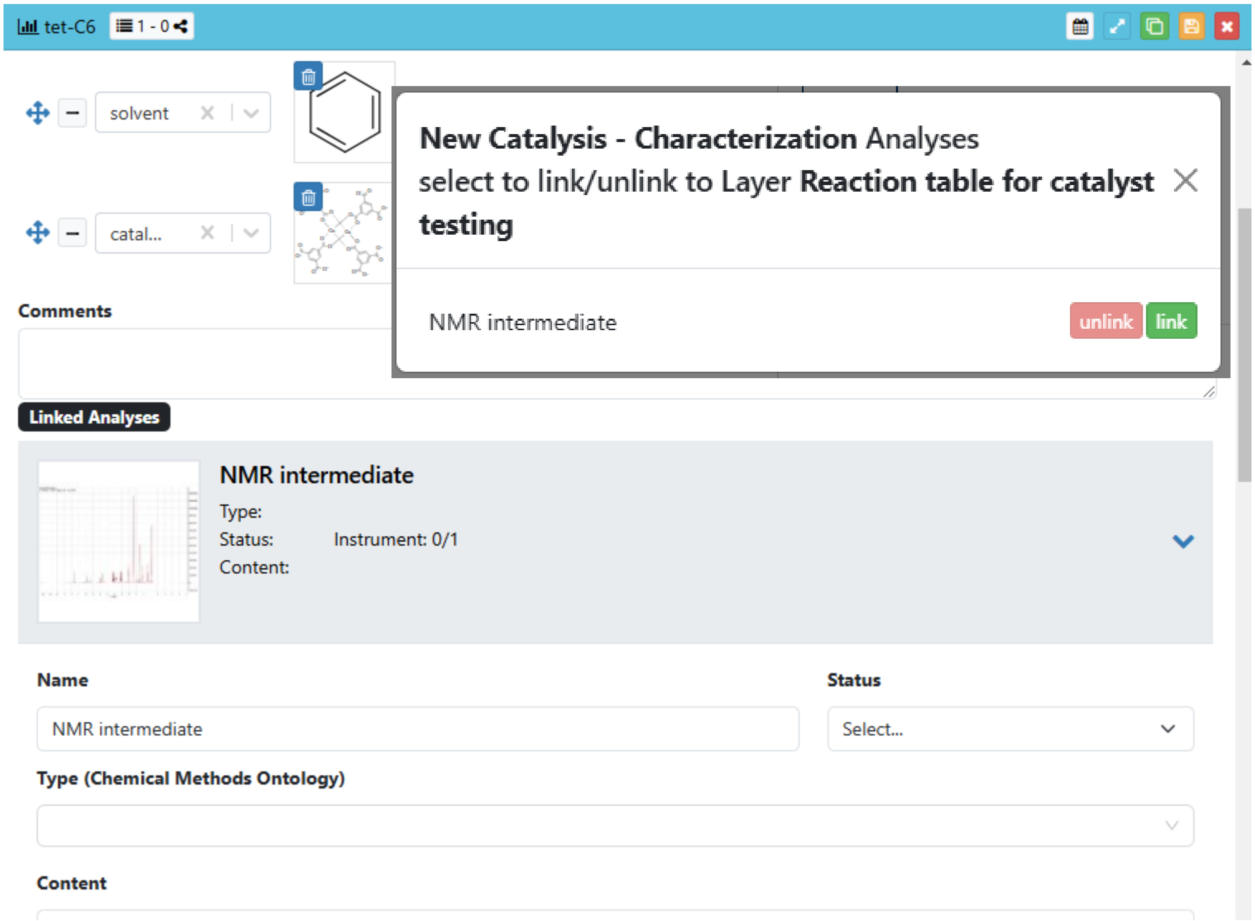

Figure S17. Screenshots showing the selection of a dataset for a linkage to a certain layer within the properties tab of generic elements.

5.2 Workflow for Element

LabIMotion assists scientists in creating customized modules tailored to their requirements. Within these modules, scientists can outline processes comprising a sequence of actions/steps aimed at achieving specific outcomes. Additionally, LabIMotion facilitates the construction of workflow logics using interactive diagrams.

A predefined workflow can be embedded in the Element. Users do not need additional configuration to access the workflow; they simply need to follow the actual process by clicking 'Next' to proceed to the next step. Users can also view the workflow by clicking the (Workflow) button.

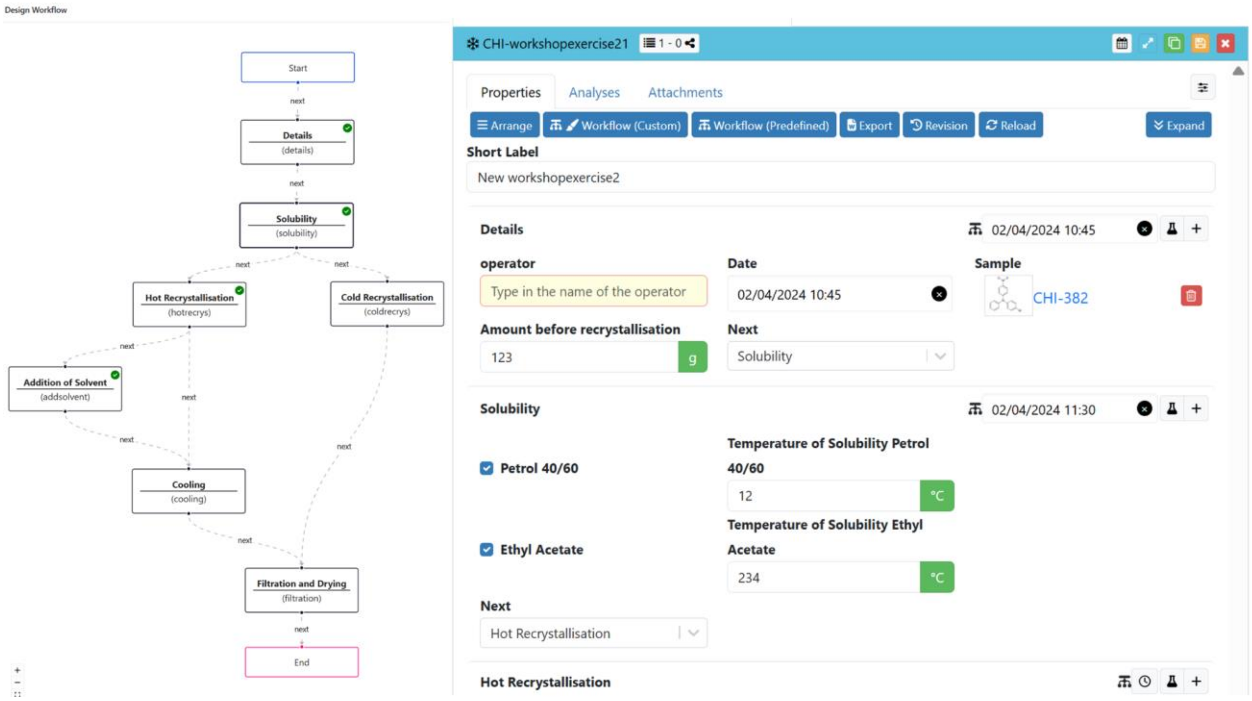

Figure S18. Representation of a workflow as workflow scheme (left) and a workflow as a form consisting of details (right). The goal is to outline processes that consist of sequential steps, providing the necessary data to achieve a specific outcome.

5.3 Dataset Metadata

Generic Dataset can also serve as a metadata vault, automatically extracting metadata from raw files and mapping it.

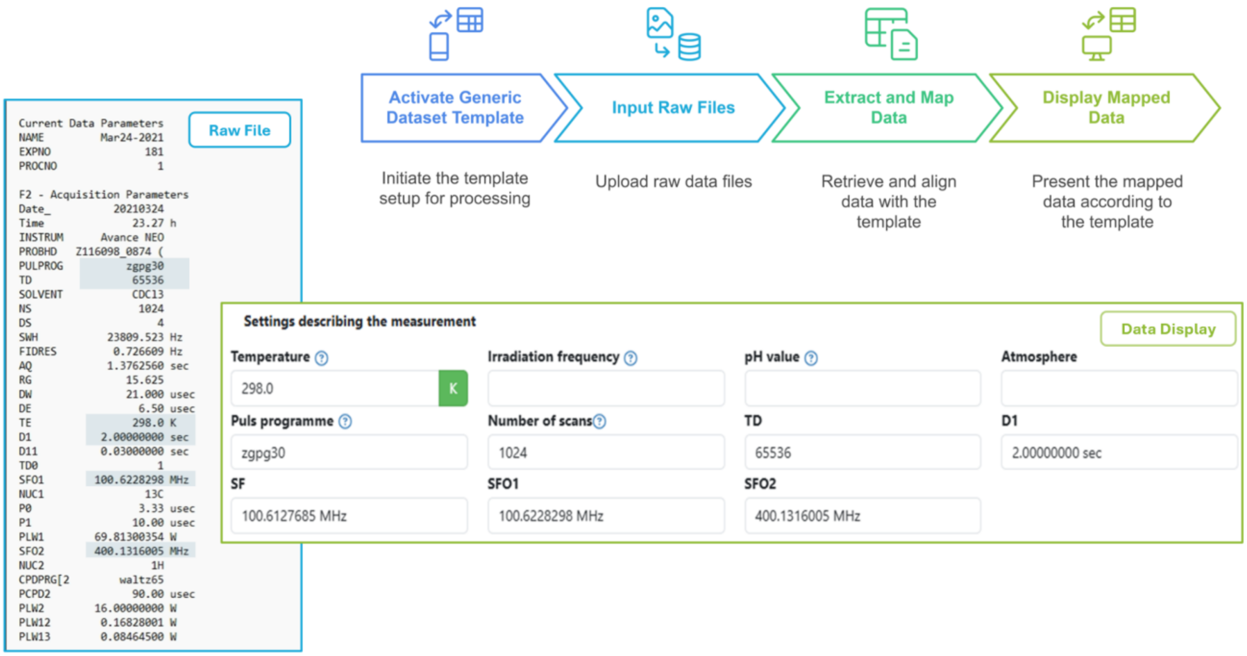

Figure S19. Extraction and mapping of metadata from a raw data file to the metadata scheme generated with a LabIMotion generic dataset. The example was taken from an 13C NMR measurement.

5.4 Export and Import Collection

LabIMotion provides a feature that allows users to export a collection containing Generic Elements, Segments and Datasets from one instance and import it into another instance when the desired template is available on both instances.

In the following scenarios, users can export/import collections containing Generic Elements, Segments and Datasets using the Export/Import Collection feature:

- Users can export/import collections containing Generic Elements, Segments and Datasets, whether within the same instance or across instances, when the templates used are synced from the Template Hub.
- Users can export/import collections containing Generic Elements, Segments and Datasets within the same instance when the templates used are synced from the Template Hub or created in the same instance.

5.5 Search Elements

The search function provides users with the ability to search for Elements. Given the diverse applications supported, the user interface for searching is designed to mirror the detailed layout of Elements. This consistency allows users to define more precise search criteria in a familiar format. Substring searching applies to “Name” and “Short Label”, which are the default fields for all Elements. For other fields, full text is required to perform a search.

5.6 Element Counter

The Element Counter function of Generic Elements gives each Element its own serial number for better organization of data. Counters can be updated manually if needed.

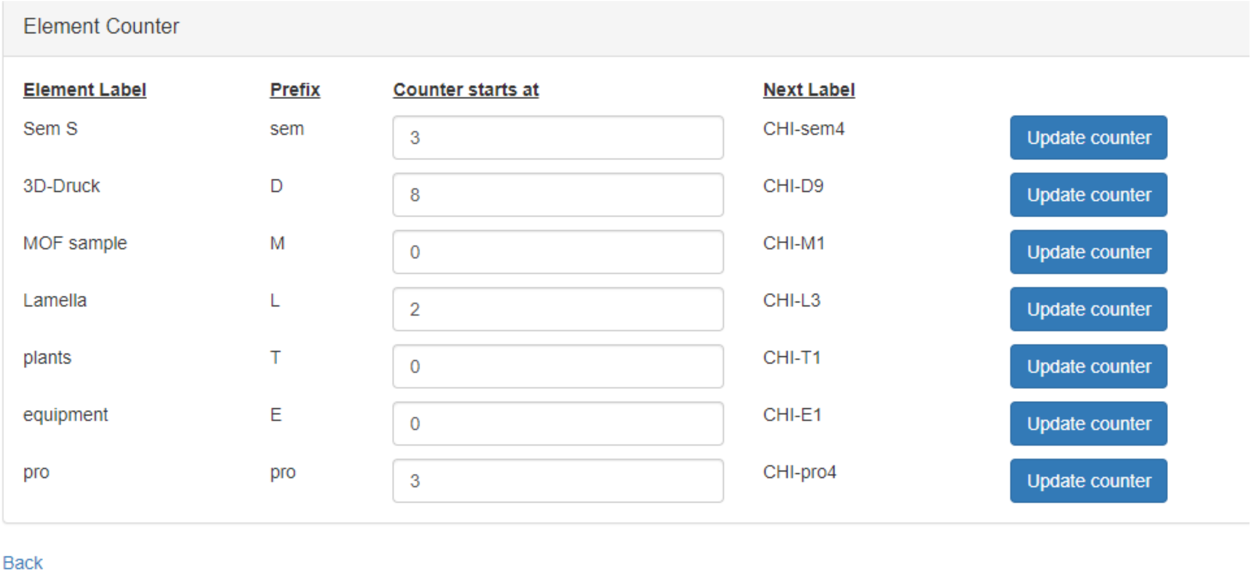

Figure S20. Screenshot showing the counter function for elements.

5.7 Revision for Element and Segment

The Revision function provides a valuable safety net that allows users to maintain the integrity of their work, track changes over time, and ensure that unexpected or unwanted changes can be easily fixed. When changes are made to content, a record of each version is kept. The Revision function includes:

- View this version: View the specific version.

- Restore Changes: Users can choose to revert to the specific version. This action restores the data to the selected version, effectively undoing any changes made after that point in time.
- Delete: If the old version is no longer needed, it can be deleted. This action is irreversible and cannot be undone once completed.

1. Potvin, C., Simon, J. & Strain, B. Effect of low temperature on the photosynthetic metabolism of the C4 grass Echinochloa crus-galli. *Oecologia* **69**, 499–506 (1986).
2. Potvin, C., Lechowicz, M. J. & Tardif, S. The statistical analysis of ecophysiological response curves obtained from experiments involving repeated measures. *Ecology* **71**, 1389–1400 (1990).
